# Cetyltrimethylammonium Bromide/Chloride on Gold Nanocrystals Can Be Directly Replaced with Tri-Citrate

**DOI:** 10.1021/acsnano.5c13995

**Published:** 2025-12-03

**Authors:** Kei Kwan Li, Ho Chang Song, Lang Xu, Yong Ding, Manos Mavrikakis, Younan Xia

**Affiliations:** † School of Chemistry and Biochemistry, 1372 Georgia Institute of Technology, Atlanta, Georgia 30332, United States; ‡ Department of Chemical and Biological Engineering, 5228 University of Wisconsin−Madison, Madison, Wisconsin 53706, United States; § 256693 School of Materials Science and Engineering, Georgia Institute of Technology, Atlanta, Georgia 30332, United States; ¶ The Wallace H. Coulter Department of Biomedical Engineering, Georgia Institute of Technology and Emory University, Atlanta, Georgia 30332, United States

**Keywords:** ligand exchange, gold nanocrystals, adsorption, surface binding, colloidal stability

## Abstract

This work demonstrates an effective method for directly exchanging the toxic cetyltrimethylammonium bromide/chloride (CTAB/C) on Au nanocrystals with tri-citrate. Our experimental and computational studies indicate that counterion plays a vital role in the exchange process. Specifically, when citrate species bind to Au surface, they all evolve into tri-citrate with different counterions. In the case of three H^+^ counterions, tri-citrate could readily replace the CTAB/C due to a strong binding of the carboxylate group with the Au surface. The substitution of H^+^ counterion by Na^+^ or K^+^ weakens the binding strength and thus compromises the exchange. Additionally, our quantitative measurements and theoretical calculations indicate that Au nanospheres encased by high-index facets are advantageous over their counterparts enclosed by {111} and/or {100} facets for the exchange owing to the difference in binding strength. The mechanistic insights and experimental control should be extendable to other combinations of surface ligands and metal nanocrystals.

## Graphical abstract

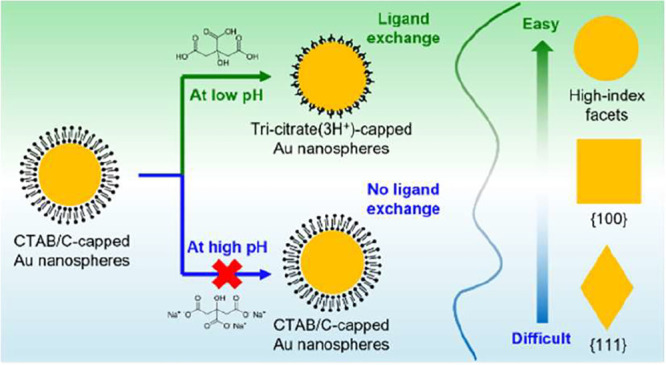

Gold (Au) nanocrystals have found widespread use in biomedical applications, including sensing, imaging, therapy, and drug delivery.^1–5^ They have been synthesized with many diverse shapes by manipulating the experimental parameters, including temperature, surface ligand, and the type and concentration of precursor or reducing agent, among others, typically involved in a colloidal synthesis.^6–11^ Among these parameters, the surface ligand plays a particularly important role by acting as a colloidal stabilizer to prevent agglomeration during both synthesis and storage while serving as a capping agent to help control shape evolution.^12,13^ However, certain ligands, as exemplified by cetyltrimethylammonium bromide/chloride (CTAB/C), adversely impact the intended biomedical application due to their inherent toxicity. To address this issue, ligand exchange must be conducted to replace the original ligands with a more suitable one without altering or compromising the properties of the nanocrystals. Despite progress, ligand exchange still faces some challenges.^12^ For example, the new ligand must have a stronger binding to the surface than the original one; otherwise, it would be difficult to achieve complete exchange. Additionally, agglomeration tends to occur during ligand exchange if the two ligands involved have opposite charges.

Citrate species are widely considered the surface ligands of choice for nanocrystals to be applied to biomedicine due to their high biocompatibility and the ability to ensure colloidal stability.^14–19^ Several protocols have been reported in the literature for effectively replacing the original ligands on nanocrystals with citrate species.^14,15^ For instance, our group developed a method involving the deposition of an ultrathin Ag layer on Au nanospheres to effectively replace the surface-bound CTAB/C with poly­(vinylpyrrolidone). Followed by etching of the Ag layer in the presence of sodium tri-citrate, we could introduce tri-citrate to the surface without changing the size and optical properties of the nanospheres.^14^ We further demonstrated that deposition of a monolayer of Au instead of Ag also resulted in effective ligand exchange without potentially contaminating the Au nanospheres with residual Ag.^15^ In addition, Yin and co-workers reported that deposition of cuprous oxide on CTAB-capped Au nanoparticles effectively removed the original ligand. In a subsequent step, Pluronic F-127 was immobilized on the particles after etching away the cuprous oxide shell.^20^ The ligand removal, however, might cause the particles to agglomerate prior to the introduction of a new ligand. As anticipated, all of these modifications led to the production of Au nanoparticles with reduced cellular toxicity, but the involvement of multiple steps inevitably caused sample loss during the exchange process.

The issue of sample loss motivates us to search for a more effective method capable of achieving direct ligand exchange in a single step. So far, direct exchange of surface ligands with citrate species is still limited, owing to two technical challenges. First, only a few studies have been conducted to examine the binding strength of citrate species to the Au surface and the explicit mechanism of ligand–surface coordination remains elusive.^21–23^ This complicates the selection of an appropriate citrate species for direct ligand exchange. Second, most Au nanocrystals are synthesized with CTAB/C as the ligands,^24–27^ which confer a positive charge to the Au surface. It is documented that directly mixing CTAB/C-capped Au nanocrystals with sodium tri-citrate would induce particle agglomeration due to the charge screening effect, significantly altering the optical properties of the sample.^28–30^

Herein, we report a systematic study to demonstrate the ability to directly exchange the CTAB/C on the surface of Au nanocrystals with citric acid in an aqueous medium. After ligand exchange, we can obtain different citrate species on the surface by simply adjusting the pH of the dispersion medium. Integrated with computational studies, we further analyze the binding between various citrate species and the surface of Au nanocrystals, a topic that remains elusive to the field of surface chemistry.^31^ Specifically, citrate species exist in different forms in an aqueous solution depending on the pH.^32^ As shown in Figure S1, four distinctive forms of citrate species bearing different charges are formed as the pH is varied: citric acid at pH = 2, mono-citrate with one counter cation at pH = 4, bi-citrate with two counter cations at pH = 5.8, and tri-citrate with three counter cations at pH = 8. Interestingly, when these citrate species bind to Au surface, they all evolve into surface-bound tri-citrate with different combinations of H^+^/Na^+^ counterions depending on the pH value (Table 1). Through experimental and density functional theory (DFT) studies involving Au nanocrystals with different shapes, we also elucidate the effects of facet type on the exchange of surface-bound CTAB/C with free citric acid in the medium. The Au nanocrystals passivated by citrate species are expected to find widespread use in biomedicine, including drug delivery, sensing, and imaging. It is also anticipated that the mechanistic insights are extendable to exchange protocols involving other pairs of ligands and metal surfaces.

**Table 1:** Summary of Citrate Species in the Dispersion Medium and on the Au Surface when the Ligand Exchange Was Performed at Different pH Values

pH value	citrate species in the solution	tri-citrate binding to Au surface
2	citric acid	tri-citrate(3H^+^)
4	sodium mono-citrate	tri-citrate(2H^+^1Na^+^)
5.8	sodium bi-citrate	tri-citrate(1H^+^2Na^+^)
8	sodium tri-citrate	tri-citrate(3Na^+^)

## Results and Discussion

### Direct Ligand Exchange of the CTAB/C on 10-nm Au Spheres with Citric Acid

The ligand exchange was conducted by directly adding an aqueous suspension of CTAB/C-capped 10-nm Au spheres into aqueous citric acid at a final concentration of 10 mM. The pH of the mixture was set to 2 to ensure that the free citric acid remained in the fully protonated form. We monitored the extent of ligand exchange by leveraging the structural differences between the chemical species and characterizing the samples obtained at different stages of a standard process for ligand exchange using Fourier transform infrared (FTIR) spectroscopy. As shown in Figure 1A, free citric acid exhibited two peaks around 1200 and 1700 cm^–1^_,_ whereas free sodium tri-citrate gave two peaks around 1400 and 1600 cm^–1^. Both peaks corresponded to the vibrational modes of carboxyl or carboxylate groups (C–O and C=O stretching, respectively). In the following discussion, these two sets of dual peaks are denoted ν-COOH for carboxyl group and ν-COO­(Na^+^) for carboxylate group, respectively, with the ion in the parenthesis corresponding to the counterion. In contrast, the FTIR spectra of CTAB/C showed characteristic doublet peaks around 2900 cm^–1^, corresponding to the stretching of CH_2_ groups (ν-CH_2_) in a long hydrocarbon chain. Figure 1A also shows the FTIR spectra recorded from the 10-nm Au spheres obtained at different stages of ligand exchange. At *t* = 0 min, the spectrum (purple trace) showed only the ν-CH_2_ peaks around 2900 cm^–1^, indicating the presence of CTAB/C on the surface. Exchange then proceeded rapidly, with the peaks associated with CTAB/C starting to disappear while new dual peaks around 1200 and 1700 cm^–1^ appeared in only 5 min (orange trace).

**Figure 1:**
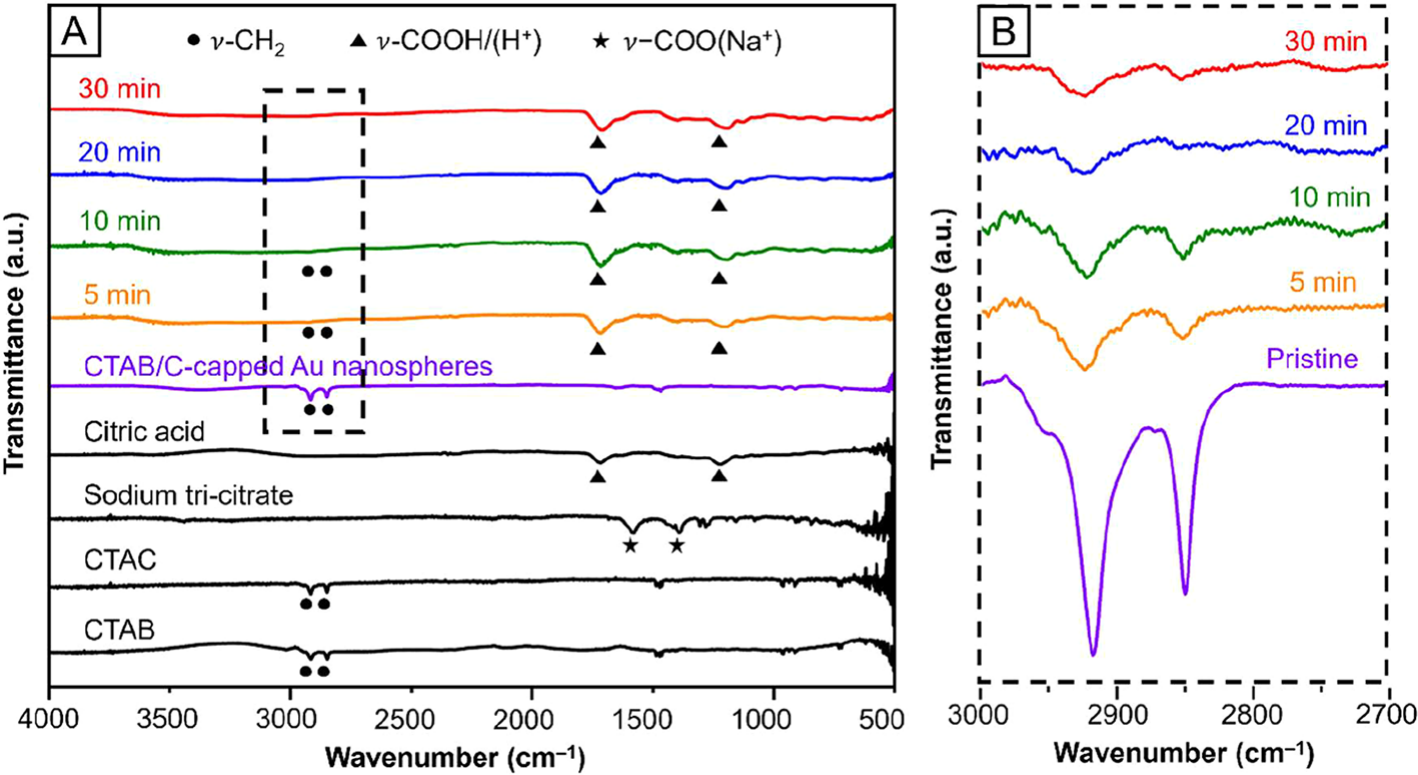
(A) FTIR spectra recorded from free citric acid, sodium tri-citrate, CTAB/C, and the 10-nm Au spheres obtained at different stages of ligand exchange with aqueous citric acid at a final concentration of 10 mM (pH = 2). (B) A segment of the FTIR spectra in the region indicated by the dashed box in (A).

Despite their similar wavenumbers to those of free citric acid, the new dual peaks had to be assigned to those of surface-bound tri-citrate with three H^+^ counterions, denoted tri-citrate­(3H^+^) hereafter, rather than citric acid where O and H are covalently bound. According to the results from our DFT calculation, the tri-citrate­(3H^+^) had a stronger binding to Au surface once the relatively weak O–H bond in the carboxyl group of citric acid had been cleaved.^33^ As such, the carboxyl group was actually deprotonated and evolved into carboxylate accompanied by a H^+^ counterion upon binding to the Au surface (Figure S2A). A detailed explanation of this binding mode, along with the exact binding energies, is provided in the last section. In this configuration, the carboxylate group consisting of C–O and C=O gave dual peaks around 1200 and 1700 cm^–1^, and these peaks should be denoted ν-COO­(H^+^) rather than ν-COOH. Specifically, the surface-bound tri-citrate­(3H^+^) and free citric acid exhibited C–O stretching peaks at 1195 and 1226 cm^–1^, respectively. The shift in wavenumber further supported that the tri-citrate­(3H^+^) was directly bound to the Au surface rather than remaining in the form of free citric acid. Otherwise, both species would exhibit the same C–O stretching frequency.

It is worth noting that the wavenumbers of the surface-bound carboxylate group differed from those reported in our previous studies, where the dual peaks were observed at 1400 and 1600 cm^–1^.^14,15^ The discrepancy could be attributed to the different counter cations involved. Specifically, the counterion alongside the surface-bound carboxylate group in the current study was H^+^, which was not expected to exert a significant impact on the binding between the carboxylate group and Au surface. In contrast, the surface-bound tri-citrate in our prior studies contained three Na^+^ counterions, denoted tri-citrate­(3Na^+^) hereafter. The Na^+^ counterion could withdraw electrons from the carboxylate group, weakening the C=O bond and thus causing a red shift, as illustrated in Figure S2B. Additionally, the Na^+^ counterion was expected to promote resonance within the carboxylate group, leading to a blue shift for the C–O bond. The shifts in wavenumbers were consistent with our calculation results, where the C=O bond of the surface-bound tri-citrate was found to decrease in wavenumbers as the counterion was switched from H^+^ to Na^+^ (Figure S3). Taken together, the counterion of the carboxylate group binding to the Au surface can have a major impact on the wavenumbers of the C–O and C=O stretching modes. The Na^+^ counterion tended to weaken the binding between citrate species and Au surface, and a detailed explanation is given in the last section.

We also took a closer look at the FTIR spectra in the region of 2700–3000 cm^–1^ (Figure 1B). Weak ν-CH_2_ peaks were still observed at *t* = 5 min, indicating the existence of some residual CTAB/C on the surface and thus incomplete exchange at this point. As ligand exchange progressed, the ν-CH_2_ peaks decreased substantially in intensity at *t* = 30 min (red trace). The gradual vanishing of the ν-CH_2_ peaks over time suggested that a sufficiently long period of time was needed to replace all of the surface-bound CTAB/C with tri-citrate­(3H^+^) although exchange could be initiated in just a few minutes. As the exchange continued, more CTAB/C would be displaced, leading to more completed ligand exchange in about 30 min.

We also measured and compared the zeta potentials of the 10-nm Au spheres before and after ligand exchange (Figure S4). The CTAB/C-capped 10-nm spheres had a zeta potential of +27.7 mV, consistent with the literature value.^14,15^ However, the zeta potentials reported for Au nanocrystals capped by citrate species corresponded to tri-citrate (3Na^+^) on the surface. In contrast, the surface ligand on the as-prepared 10-nm Au spheres was tri-citrate­(3H^+^), making it difficult to make direct comparison. To bridge this gap and enable a direct comparison, we adjusted the pH of the suspension containing the 10-nm Au spheres capped by tri-citrate­(3H^+^) from 2 to 12 by adding aqueous NaOH and thus converting the H^+^ counterion to Na^+^. At pH = 12, the Au nanospheres had a zeta potential of −31.0 mV (Figure S4), which was very close to the value of −38.4 mV reported in the literature.^34^ The negative zeta potential implied the successful replacement of the original CTAB/C by citric acid while indicating that the surface of the 10-nm Au spheres was covered by tri-citrate rather than citric acid.

Owing to the involvement of both CTAB and CTAC in the synthesis, Br^–^ and Cl^–^ were expected to be co-adsorbed on the 10-nm Au spheres prior to ligand exchange. This was confirmed by the presence of Br 3d and Cl 2p peaks in the X-ray photoelectron spectroscopy (XPS) spectra recorded from the CTAB/C-capped nanospheres (Figure S5A–C). Notably, since Br^–^ binds to the Au surface more strongly than Cl^–^,^35^ the surface-bound CTAB and CTAC would be replaced at different efficiencies. After ligand exchange for 30 min, the Cl 2p peak disappeared, whereas a small Br 3d peak was still observed (Figure S5D–F), implying that essentially all of the CTAC had been exchanged with citric acid but some residual CTAB remained on the surface. It should be pointed out that 30 min was not adequate to push the equilibrium described in Equation 1 completely to the right side, and thus a trace amount of CTAB were remained on the surface. The discrepancy between FTIR and XPS data can be attributed to their difference in detection limits, with XPS being more sensitive than FTIR for the detection of surface-bound species. In general, one can increase the completeness of the ligand exchange by elongating the incubation time.

$$\mathrm{CTAB}/C_{\mathrm{adsorbed}}+\mathrm{citric}\;{\mathrm{acid}}_{\mathrm{free}}⇌\mathrm{CTAB}/C_{\mathrm{free}}+\mathrm{tri}‐\mathrm{citrate}(3H^{+}{)}_{\mathrm{adsorbed}}$$ 1

We also quantified the number of surface-bound CTAB molecules on each 10-nm Au sphere before and after ligand exchange by measuring the Br content with inductively coupled plasma mass spectrometry (ICP-MS). The original CTAB/C-capped sample had about 1402 CTAB molecules per particle, and the number dropped drastically to 60 after ligand exchange (see the Experimental Section). As demonstrated in our prior cell culture study,^15^ such a small amount of CTAB would not cause toxicity to the cells.

Interestingly, the XPS spectra showed blue shifts for the Au 4f_7/2_ and 4f_5/2_ peaks after ligand exchange. Specifically, the binding energy of the former increased from 83.48 to 83.68 eV and that of the latter from 87.08 to 87.38 eV. Prior to ligand exchange, Br^–^ and Cl^–^ bound to the Au surface,^31,36,37^ whereas the Au atom was coordinated to the oxygen atom of the carboxylate group upon exchange. Since oxygen withdrew more electrons from Au than Br^–^ and Cl^–^, tri-citrate­(3H^+^) could withdraw more electrons from surface Au atoms than CTAB/C, leading to a higher binding energy for the Au 4f electrons. Again, the observed blue shifts to the Au 4f peaks confirmed the success of ligand exchange.

Figure S6 shows a schematic of the exchange process between surface-bound CTAB/C and free citric acid in solution. According to the literature, CTAB/C are supposed to bind to the surface of Au nanospheres in a Au–(Br/Cl)–N configuration.^31,36,37^ The tertiary ammonium headgroup of CTA^+^ does not possess any lone pair electrons that can be donated to the Au atoms on the surface. Additionally, there is steric hindrance from the methyl groups connected to the nitrogen atom, preventing the ammonium group from binding to the Au surface effectively. In contrast, the negatively charged halide ion with four lone pairs of electrons can readily be adsorbed on the surface of Au nanospheres through coordination and thus attract the ammonium group through a relatively strong electrostatic interaction. In such a configuration, the hydrophobic tail of CTA^+^ points away from the Au surface, promoting the formation of a bilayer comprised of CTAB/C when the Au nanospheres are suspended in an aqueous solution.

Upon adding the suspension of Au nanospheres into an aqueous solution of citric acid, some of the surface-bound CTAB/C were expected to be desorbed from the surface owing to the absence of free CTAB/C in the medium,^38,39^ generating voids in the bilayer. Such voids allowed the free citric acid in the solution to access the Au surface. As established by our DFT results below, tri-citrate­(3H^+^) bound to Au more strongly than CTAB/C,^31^ forcing the CTAB/C on the Au surface to be replaced by tri-citrate­(3H^+^). The replacement of some of the surface-bound CTAB/C by tri-citrate­(3H^+^) would destabilize the bilayer,^40^ creating more voids to promote further ligand exchange. As a result, the CTAB/C was continuously exchanged with citric acid, making the Au surface increasingly covered by tri-citrate­(3H^+^).

Introducing a new ligand to the surface can alter the stability of a colloidal suspension.^42^ Specifically, the new ligands may attenuate the electrostatic repulsion among the nanocrystals when they bear a charge opposite the original ligands, causing agglomeration. The agglomeration of Au nanocrystals is often accompanied by color changes to the suspension as a result of the broadening and red shift of the localized surface plasmon resonance (LSPR) peak.^41^ As shown by the UV–vis spectrum recorded from an aqueous suspension of the original CTAB/C-capped 10-nm spheres (black trace in Figure 2), a sharp LSPR peak was observed at 521 nm, and the suspension displayed a ruby-red color. The LSPR peak has been reported to broaden and red-shift upon adding aqueous sodium tri-citrate, together with a color change to purple, due to charge screening and thus the occurrence of particle agglomeration.^14^

**Figure 2:**
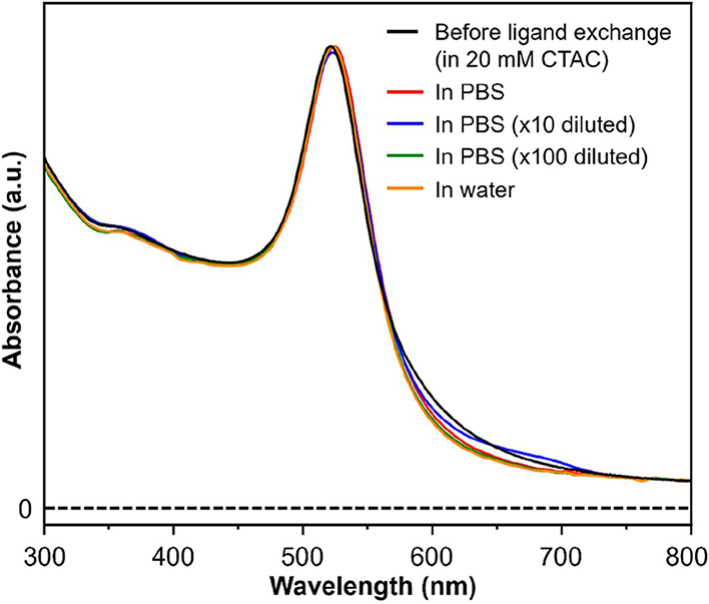
UV–vis spectra recorded from aqueous suspensions of the tri-citrate­(3H^+^)-capped 10-nm Au spheres in PBS (red), in PBS at a 10-fold dilution (blue), in PBS at a 100-fold dilution (green), and water (orange). The UV–vis spectrum recorded from aqueous suspensions of the CTAB/C-capped 10-nm Au spheres in 20 mM aqueous CTAC (black) was also included for a direct comparison of the colloidal stability.

We also examined the colloidal stability of the 10-nm Au spheres obtained through ligand exchange with citric acid using UV–vis spectroscopy. To mimic the physiological conditions associated with biomedical applications, the nanospheres capped by tri-citrate­(3H^+^) were collected by centrifugation and then suspended in phosphate-buffered saline (PBS) solution at varying concentrations, as well as deionized water (pH = 7). As shown in Figure 2, all of the aqueous suspensions of the tri-citrate­(3H^+^)-capped 10-nm Au spheres exhibited similarly narrow LSPR peak, together with retention of the peak position, when compared to the original CTAB/C-capped sample (black traces). This data suggested that the colloidal stability of the 10-nm Au spheres was maintained in media with varying ionic strengths after ligand exchange.^40^ Specifically, the surface-bound tri-citrate­(3H^+^) was able to provide a strong electrostatic repulsion among the particles to stabilize the colloidal suspension. In addition, it was reported that the citrate species on the surface of Au nanoparticles could provide steric repulsion in stabilizing the colloidal suspension.^43^ Altogether, both electrostatic and steric repulsions were expected to play a key role in preventing the particles from agglomeration. One might argue that the ionic species in PBS would screen the surface charges and thus induce agglomeration. However, it should be noted that approximately 95% of the ions in PBS were mono-charged, which were less effective than the free tri-charged citrate ions in screening the charges on the surface of the particles.

Long-term colloidal stability is one of the key parameters in determining the functionality of nanocrystals,^44^ particularly in biomedical applications that require on-shelf storage or transportation. To evaluate long-term colloidal stability, we also recorded UV–vis spectra from all the suspensions of the tri-citrate­(3H^+^)-capped 10-nm Au spheres after storage at room temperature over a period of 1 week (Figure S7). The LSPR peak remained essentially unchanged during storage in terms of both the peak width and position, indicating that the colloidal stability was well retained. The results demonstrated excellent colloidal stability of the tri-citrate­(3H^+^)-capped 10-nm Au spheres under ambient conditions.

One of the fascinating properties associated with the Au nanospheres is the possession of a highly symmetric shape and thus a single narrow LSPR peak^45,46^ and sensitive color changes in sensing applications such as lateral flow assay. As reported in the literature,^47^ replacing the original ligand on the high-index facets of nanocrystals with a new one might induce surface reconstruction and thus shape transformation. Although we did not observe any major change to the LSPR peak, we still employed transmission electron microscopy (TEM) analysis to confirm the perseverance of the spherical shape during the exchange process. Figure 3A shows a TEM image of the CTAB/C-capped 10-nm Au spheres, with an average diameter of 10.0 ± 0.4 nm. As shown in Figure 3B, both the size and spherical shape taken by the original Au nanospheres were retained during ligand exchange, confirming that treatment with citric acid did not lead to significant surface reconstruction.

**Figure 3:**
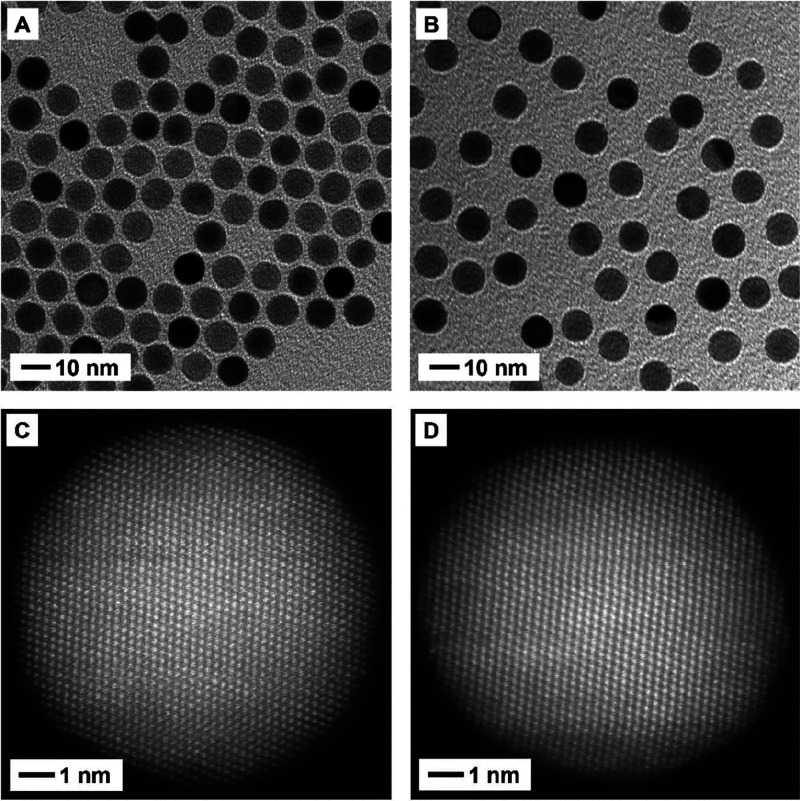
(A, B) TEM and (C, D) HAADF-STEM images of the 10-nm Au spheres before and after ligand exchange for 30 min with aqueous citric acid at a final concentration of 10 mM (pH = 2): (A, C) CTAB/C-capped and (B, D) the corresponding tri-citrate­(3H^+^)-capped 10-nm Au spheres, respectively.

We further confirmed the preservation of high-index facets by high-angle annular dark-field scanning transmission electron microscopy (HAADF-STEM) analysis. Figure 3C,D shows two images recorded from different 10-nm Au spheres in the same batch of sample, before and after the ligand exchange, respectively, along the [011] zone axis. Both particles showed a nearly perfect spherical shape, containing similar steps, kinks, and high-index facets that are characteristic of a spherical particle. Taken together, the ligand exchange did not alter the spherical shape of the 10-nm spheres, as supported by the consistent results obtained from UV–vis spectroscopy, TEM, and HAADF-STEM analyses.

To demonstrate the universality of the method, we extended the ligand exchange to 20- and 60-nm Au spheres (Figure S8A–D). Before ligand exchange, the FTIR spectra of both samples showed ν-CH_2_ peaks around 2900 cm^–1^, indicating the presence of CTAB/C on the surface (Figure S8E). We then recorded FTIR spectra of the nanospheres after ligand exchange for 30 min, as depicted by the green and purple traces in Figure S8E for the 20- and 60-nm spheres, respectively. Both spectra displayed strong ν-COO­(H^+^) peaks around 1200 and 1700 cm^–1^ and the disappearance of ν-CH_2_ peaks, implying a successful replacement of CTAB/C by tri-citrate­(3H^+^). Zeta potential measurements further supported the successful ligand exchange. As shown in Figure S8F, the 20- and 60-nm spheres after ligand exchange exhibited zeta potentials of −31.9 and −34.3 mV at pH = 12, respectively, which were comparable to what was obtained for the 10-nm Au spheres. Altogether, the FTIR and zeta potential analyses suggested that the protocol for ligand exchange is applicable to Au nanospheres with diameters ranging from 10 to 60 nm. It should be noted that the shape of nanocrystals may influence the efficiency of ligand exchange due to the presence of different facets on the surface, and a more detailed study can be found in a later section.

### Effect of Counter Cations on Ligand Exchange

As shown in Figure S1, citrate species can be found in multiple forms that bear different numbers of counter cations as a function of pH. Following the standard protocol, we also adjusted the pH of the mixture from 2 to 4, 5.8, and 8 by adding different amounts of aqueous NaOH (see the Experimental Section) to investigate the effect of Na^+^ counterion on ligand exchange. These pH values corresponded to the dominance of mono-citrate, bi-citrate, and tri-citrate with one, two, and three Na^+^ counterions, respectively, in the solution. We then used FTIR spectroscopy to analyze the identities of the surface ligands on the 10-nm Au spheres after ligand exchange using the standard protocol at the specific pH. As shown in Figure 4, the surface-bound CTAB/C could be fully replaced by tri-citrate­(3H^+^) in 30 min at pH = 2 (red trace).

**Figure 4:**
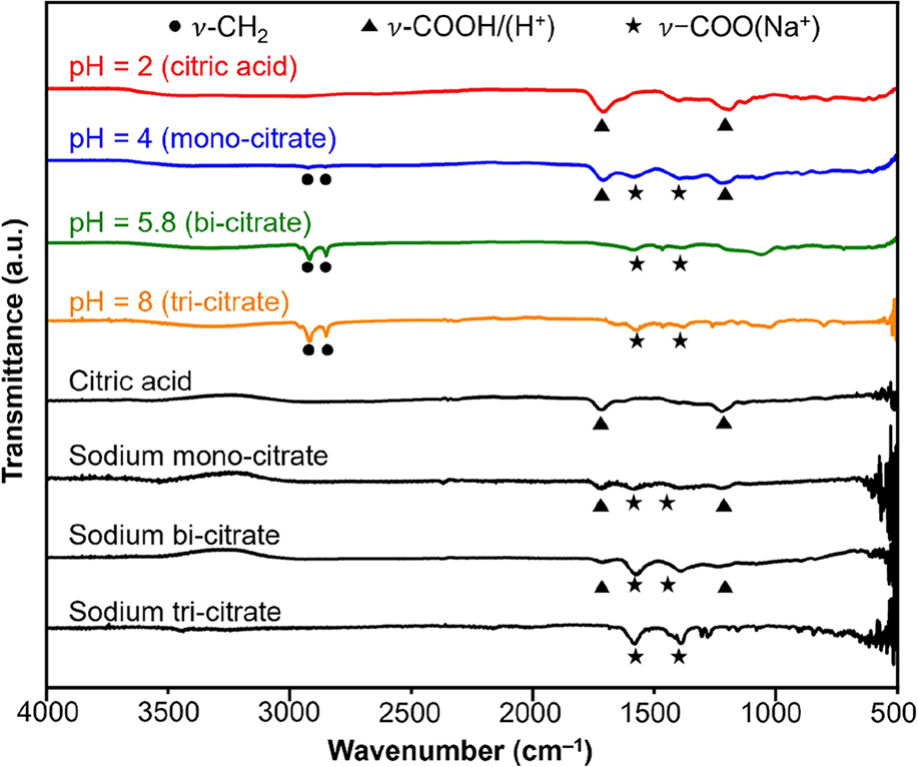
FTIR spectra recorded from the references (free sodium tri-citrate, sodium bi-citrate, sodium mono-citrate, and citric acid) and 10-nm Au spheres obtained through ligand exchange at different pH for 30 min. The dominant citrate species in the solution at different pHs are indicated in the parenthesis.

At pH = 4, mono-citrate became the dominant species in the medium, and the FTIR spectrum (blue trace) displayed prominent ν-COO­(H^+^) peaks, together with ν-COO­(Na^+^) peaks in the region of 1400–1600 cm^–1^. It should be noted that the Na^+^ counterion could alter the binding between the carboxylate groups and Au surface as illustrated in Figure S2B. Accordingly, two sets of peaks associated with the carboxylate group containing H^+^ and Na^+^ counterions were observed in the FTIR spectrum. Overall, the simultaneous presence of ν-COO­(H^+^) and ν-COO­(Na^+^) peaks confirmed that mono-citrate could also substitute CTAB/C and be adsorbed onto the surface of 10-nm Au spheres during the ligand exchange process. However, weak doublet peaks were still observed around 2900 cm^–1^, indicating that some of the surface-bound CTAB/C could not be replaced when the incubation time was 30 min only. The coexistence of ν-COO­(H^+^), ν-COO­(Na^+^), and ν-CH_2_ peaks confirmed that ligand exchange could not be completed in 30 min at pH = 4.

Ligand exchange did not proceed effectively at pH = 5.8 and 8, when bi-citrate and tri-citrate, respectively, were the dominant species in the medium. Specifically, the ν-COO­(H^+^) peaks completely vanished, while the ν-COO­(Na^+^) peaks decreased substantially in intensity compared to the sample at pH = 4, and ν-CH_2_ peaks were still observed around 2900 cm^–1^. The FTIR spectra suggested that the surface of the 10-nm Au spheres remained largely covered by CTAB/C, with negligible amounts of tri-citrate­(1H^+^2Na^+^) or tri-citrate­(3Na^+^) on the surface, indicating low efficiency in ligand exchange at pH = 5.8 and 8. The presence of weak ν-COO­(Na^+^) peaks could be ascribed to *i*) the replacement of a small portion of surface-bound CTAB/C with tri-citrate­(1H^+^2Na^+^) or tri-citrate­(3Na^+^) and/or *ii*) the electrostatic trapping of some tri-citrate­(1H^+^2Na^+^) or tri-citrate­(3Na^+^) anions on the surface due to the attraction from the CTA^+^ cation.

To further confirm the effect of counter cations on ligand exchange, we repeated the experiment using KOH instead of NaOH. As shown in Figure S9, the FTIR spectra of the 10-nm Au spheres after ligand exchange displayed ν-CH_2_ peaks in all cases when pH = 4, 5.8, or 8, which was consistent with what was obtained using NaOH. The different behaviors between various citrate species involved in the ligand exchange process can be attributed to their differences in binding strength to the Au surface. The carboxylate group accompanied by the H^+^ counterion exhibited stronger binding to the Au surface relative to CTAB/C, while replacing the H^+^ with Na^+^ or K^+^ greatly reduced the binding strength. The difference in binding strength favored ligand exchange when adsorbates were based on tri-citrate­(3H^+^).

To gain insights into the influence of counter cations on the binding strength of citrate species to the surface of 10-nm Au spheres, we used UV–vis spectroscopy to monitor the adsorption/desorption behavior of tri-citrate­(3H^+^) and tri-citrate­(3Na^+^) on 10-nm Au spheres upon dilution. Specifically, we first prepared 10-nm Au spheres capped by tri-citrate­(3H^+^) and tri-citrate­(3Na^+^) and subsequently suspended them in 0.01 M of aqueous citric acid (pH = 2) and aqueous sodium tri-citrate (pH = 12), respectively. The former sample was prepared in the current work. However, the same condition for ligand exchange was proven ineffective for the latter case, and we had to follow a previously reported protocol that involved the deposition of a Au monolayer to replace the surface-bound CTAB/C with tri-citrate­(3Na^+^).^15^ As noted at the end of this section, it was also impossible to obtain tri-citrate­(3Na^+^)-capped Au nanospheres from the tri-citrate­(3H^+^)-capped sample by simply increasing the pH due to the involvement of agglomeration. To ensure a fair comparison, we kept the concentration (6.2 × 10^10^ particles/mL), particle size (10 nm), and ionic strength similar for both samples. The aqueous suspensions of both samples were then diluted to different volumes and changes to the LSPR peak width and position were analyzed to probe possible agglomeration. The dilution of tri-citrate­(3H^+^)-capped 10-nm Au spheres was performed with 0.01 M HCl to maintain a constant pH of 2 for the suspension, thereby eliminating any possible structural changes to the surface-bound tri-citrate­(3H^+^) and free citric acid that could induce agglomeration. For the tri-citrate­(3Na^+^)-capped sample, it was diluted with 0.01 M NaOH to maintain a similar ionic strength as the tri-citrate­(3H^+^)-capped counterpart.

Figure 5A,B shows UV–vis spectra recorded from the two samples upon dilution. The LSPR peak width and position were identical for both samples when the particles were freshly suspended in 0.01 M aqueous citric acid or sodium tri-citrate (red trace in Figure 5 A,B, respectively), indicating good colloidal stability for the aqueous suspensions. For the tri-citrate­(3H^+^)-capped 10-nm Au spheres, the width and position of their LSPR peak remained essentially unchanged when the suspension was diluted in volume by 2- and 10-fold (the blue and green traces in Figure 5A, respectively). However, a significant broadening of the LSPR peak was observed when the suspension was diluted by 100-fold (the orange trace in Figure 5A), indicating particle agglomeration under this condition. In contrast, the LSPR peak associated with the aqueous suspension of tri-citrate­(3Na^+^)-capped 10-nm Au spheres exhibited notable broadening in the case of only a 2-fold dilution (the blue trace in Figure 5B). Further dilution by 10- and 100-fold resulted in more pronounced broadening and even a red shift for the LSPR peak (the green and orange traces in Figure 5B, respectively), suggesting severe agglomeration of the particles. Altogether, the tri-citrate­(3H^+^)-capped 10-nm Au spheres demonstrated greater resistance against agglomeration during dilution when compared with the counterpart involving tri-citrate­(3Na^+^). This discrepancy in stability can be attributed to the fact that tri-citrate­(3H^+^) binds to Au surface more strongly than tri-citrate­(3Na^+^) and is thus less susceptible to desorption from the surface upon dilution.

**Figure 5:**
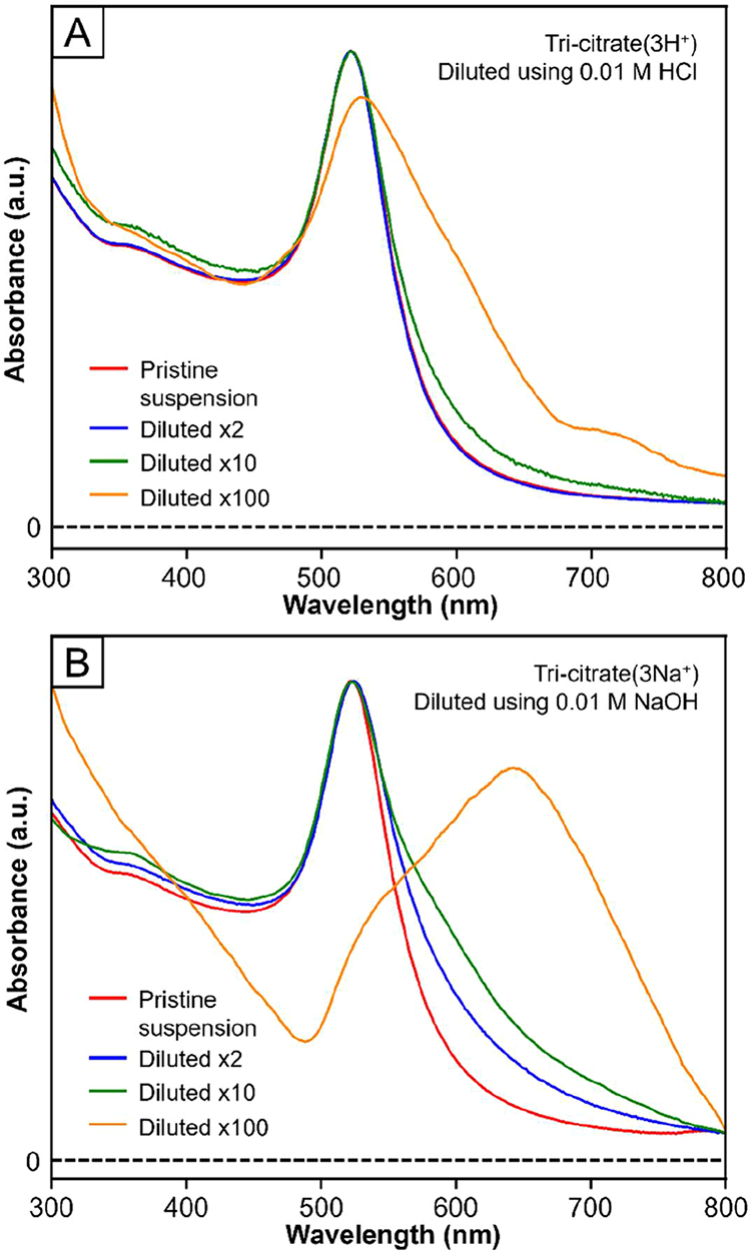
UV–vis spectra recorded from the aqueous suspension of the 10-nm Au spheres capped by (A) tri-citrate­(3H^+^) and (B) tri-citrate­(3Na^+^) in aqueous citric acid (pH = 2) and sodium tri-citrate solution (pH = 12), respectively, at a concentration of 0.01 M. UV–vis spectra recorded from the same samples upon dilution by different factors (blue: by 2-fold, green: by 10-fold, and orange: by 100-fold). All spectra were normalized to the peak intensity for easy comparison.

We further evaluated the colloidal stability of each suspension in Figure 5 by recording the UV–vis spectra over a period of 48 h (Figure S10). For the pristine suspension, both tri-citrate­(3H^+^)- and tri-citrate­(3Na^+^)-capped nanospheres showed stable LSPR peak with no broadening or shift after 48 h, suggesting good colloidal stability (Figure S10A,B). Upon 2-fold dilution, only the tri-citrate­(3H^+^)-capped nanospheres maintained a sharp LSPR peak at *t* = 48 h, while the tri-citrate­(3Na^+^) counterpart began to show peak broadening as early as at *t* = 24 h due to agglomeration (Figure S10C,D). The difference in colloidal stability further confirmed that tri-citrate­(3H^+^) binds more strongly to the Au surface than tri-citrate­(3Na^+^). Both the 10-nm Au spheres showed agglomeration at *t* = 24 h at a higher dilution factor of 10 or 100, as indicated by the broadening and red shift of the LSPR peak (Figure S10E–H). The changes were more pronounced in the tri-citrate­(3Na^+^)-capped nanospheres. Taken together, it can be concluded that tri-citrate­(3H^+^) served as a better ligand to prevent the 10-nm Au spheres from agglomeration due to its stronger binding to the Au surface than tri-citrate­(3Na^+^).

The difference in agglomeration behavior between the tri-citrate­(3H^+^)- and tri-citrate­(3Na^+^)-capped 10-nm Au spheres upon dilution can be explained using the Langmuir model.^37,38^ According to this model (Figure S11), the ligands are expected to be desorbed from the surface of 10-nm Au spheres in a medium lacking free citric acid or tri-citrate (i.e., with *C*, which is the concentration of free ligands, approaching zero), thereby reducing their surface coverage (θ). Once the θ-value drops below a threshold for agglomeration (θ_agglomeration_), the remaining ligands are no longer adequate to stabilize the nanospheres, causing agglomeration. In the current study, the two samples of 10-nm Au spheres, one capped by tri-citrate­(3H^+^) and the other by tri-citrate­(3Na^+^), had the same diameter and hence the same surface area. They should share the same θ_agglomeration_. As shown by the UV–vis spectra in Figure 5, the tri-citrate­(3H^+^)-capped nanospheres exhibited a retarded tendency toward agglomeration upon dilution compared to their tri-citrate­(3Na^+^) counterpart, suggesting that they reached θ_agglomeration_ at a lower *C* (Figure S11). These trends further indicated that tri-citrate­(3H^+^) had a larger binding constant (*K*) in the Langmuir model than tri-citrate­(3Na^+^), implying stronger binding to the Au surface. This result suggested that the Na^+^ counterion tended to weaken the binding of the carboxylate group to the surface of 10-nm Au spheres. To understand the destabilizing effect of the Na^+^ counterion on the binding, we performed DFT calculations to elucidate the atomic-scale interactions between citrate species with different types of Au facets in the presence or absence of Na^+^ counterions (see the next section).

It is worth pointing out that our current understanding of the influence exerted by Na^+^ counterions on the binding of carboxylate group to the Au surface is still qualitative as the exact *K* values for the adsorption of tri-citrate­(3H^+^) and tri-citrate­(3Na^+^) on the Au surface cannot be found in the literature. At the moment, the θ_agglomeration_ in the Langmuir model remains unknown, making it impossible to determine the exact *K* value by substituting the experimental data into the model. In addition, we can only use a simple model built upon a homogeneous surface, where the adsorbed species do not dissociate and there is no interaction between the adsorbed ligands. Altogether, the conclusion regarding the binding strength of different citrate species to the Au surface is still qualitative rather than quantitative.

In addition to the unsuccessful replacement of CTAB/C by tri-citrate containing Na^+^ counterion, we observed gradual agglomeration of the 10-nm Au spheres during ligand exchange as the pH was increased, as revealed by the broadening and red shift of the LSPR peak (Figure S12). Specifically, increasing the pH from 2 to 4 resulted in slight broadening of the peak while the peak position remained unchanged (blue trace). At pH = 5.8, a more significant broadening, together with a slight red shift, was recorded (green trace). The changes to the LSPR peak intensified at pH = 8, where doubling of the peak width and a dramatic red shift of 100 nm were observed (orange trace). Overall, increasing the pH promoted the agglomeration of 10-nm Au spheres, which could be attributed to the presence of free bi- and tri-citrate species in the medium that significantly screened and attenuated the electrostatic repulsion among the positively charged CTAB/C-capped 10-nm Au spheres. A similar scenario of agglomeration was also reported when sodium tri-citrate was directly added into an aqueous suspension of CTAB/C-capped Au nanospheres.^14^

Taken together, the exchange of the CTAB/C on 10-nm Au nanospheres with different citrate species was only effective when the ligand of interest was tri-citrate­(3H^+^). Meanwhile, direct ligand exchange simply failed when the ligands of interest became tri-citrate­(3Na^+^). The unsuccessful ligand exchange could be ascribed to the weakened binding of carboxylate groups to the Au surface caused by the Na^+^/K^+^ counterions.

### Quantitative Analysis of the Binding between Tri-citrate­(3H^+^) and Different Au Facets

The Au nanospheres exhibit a higher abundance of high-index facets, particularly {211}, {311}, and {331}, when compared to nanocrystals in other shapes, including {111}-capped octahedra and {100}-capped cubes.^47^ The higher abundance of high-index facets seemed to play a critical role in facilitating the ligand exchange between surface-bound CTAB/C and tri-citrate­(3H^+^). Using the standard protocol, we also extended ligand exchange to {100}-capped cubic and {111}-capped octahedral Au nanocrystals of 21 nm in edge length (Figure S13A,B). As shown by the TEM image in Figure S13A, some particles in the sample of cubic nanocrystals exhibited a bipyramidal or rod-like shape, likely due to the fast reduction kinetics involved in the growth and the templating effect arising from the singly- or multiply twinned seeds. However, all these nanocrystals were enclosed by {100} facets, and hence, the difference in shape was not expected to exert a major impact on the ligand exchange. The variations in projected profiles observed in Figure S13B could be attributed to octahedral nanocrystals with different orientations relative to the electron beam. Figure S13C,D shows TEM images of the nanocrystals after the ligand exchange for 30 min. The preservation of their original shapes indicated that their incubation with citric acid did not induce surface atomic migration or oxidative etching.^47^ As such, any observed alterations to the surface speciation could be attributed to the efficacy of ligand exchange.

Again, we employed FTIR spectroscopy to identify the ligands on the surface of the Au nanocrystals before and after the ligand exchange process. First, we obtained FTIR spectra of the CTAB/C-capped Au nanocrystals with different shapes (Figure 6A), and the strong ν-CH_2_ peaks around 2900 cm^–1^ confirmed the presence of CTAB/C on all samples. We then recorded FTIR spectra of the nanocrystals after ligand exchange, as depicted by the green, red, and blue traces in Figure 6A for 20-nm spheres, 21-nm cubes, and 21-nm octahedra, respectively. All samples showed strong ν-COO­(H^+^) peaks at around 1200 and 1700 cm^–1^, implying the occurrence of ligand exchange. However, the ν-CH_2_ peaks were only absent in the spectrum associated with the spherical nanocrystals. In contrast, the ν-CH_2_ peaks remained relatively strong in the cubic and octahedral samples (Figure 6B), indicating the presence of a significant amount of CTAB/C on the surface due to incomplete ligand exchange. It is worth noting that the ν-CH_2_ peaks in the FTIR spectrum of the octahedral nanocrystals after ligand exchange were stronger than those of the spherical and cubic samples, indicating the presence of more residual CTAB/C on the surface. Taken together, the extent of ligand exchange at *t* = 30 min decreased in the order of spheres > cubes > octahedra. The variations in ligand exchange on different nanocrystals could be attributed to the high-index facets exposed on the surface of spherical nanocrystals, and a detailed analysis can be found in the next section.

**Figure 6:**
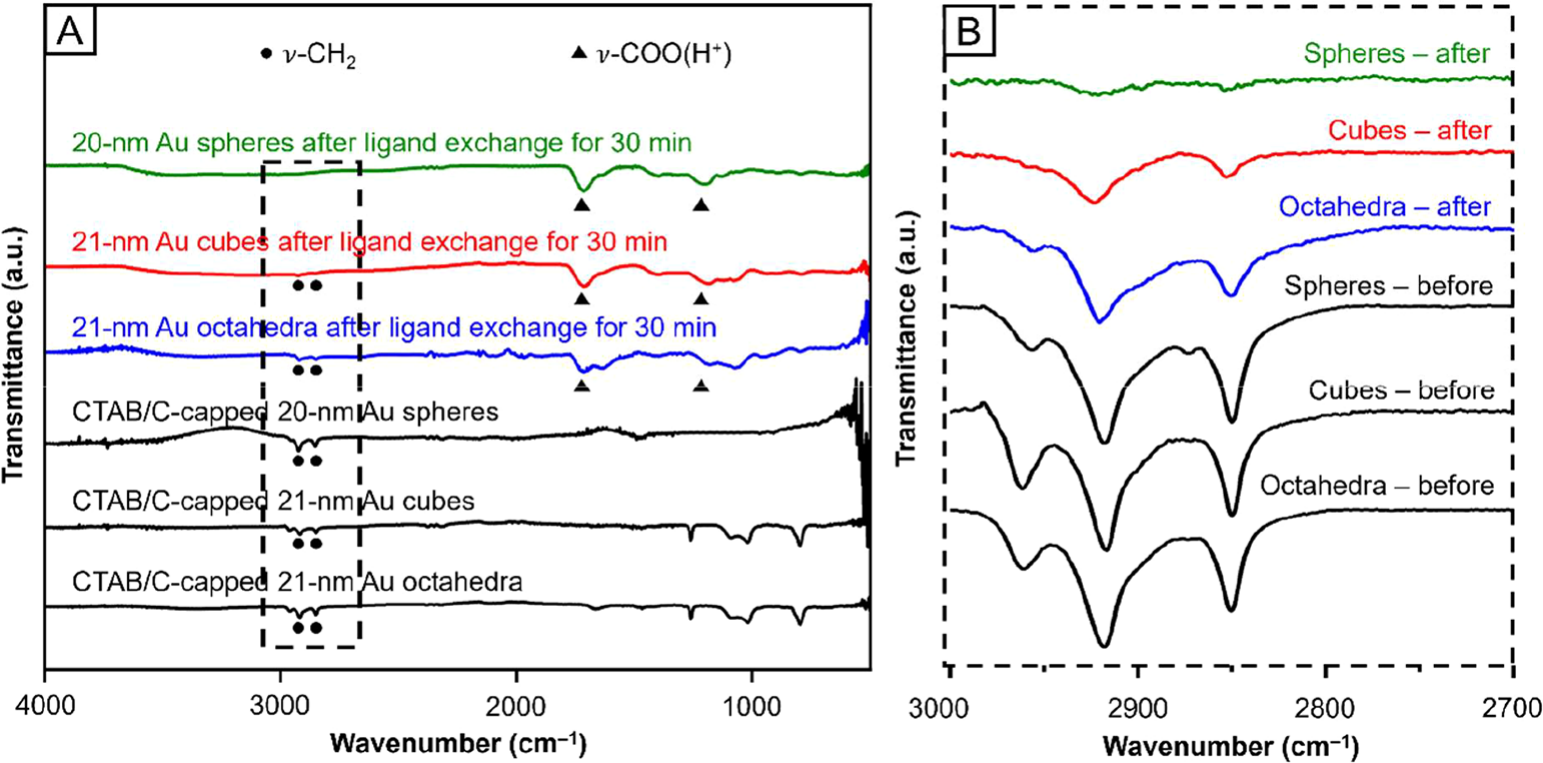
(A) FTIR spectra recorded from the 20-nm spheres, 21-nm cubes, and 21-nm octahedra before and after the ligand exchange for 30 min with aqueous citric acid at a final concentration of 10 mM (pH = 2). (B) A segment of the FTIR spectra in the region indicated by the dashed box in (A).

We also employed FTIR spectroscopy to achieve a quantitative comparison of the extents of ligand exchange on different types of nanocrystals. By applying the Beer–Lambert law, one can derive the concentration (*c*) of a compound using Equation 2:

c=A/εl 2

where *A* is the absorbance, ε is the molar attenuation coefficient of the compound, and *l* is the optical path length. It should be pointed out that FTIR spectroscopy is commonly regarded as a qualitative method when applied to analyze the surface ligands on nanocrystals due to the involvement of attenuated total reflectance and thus uncertainty about the optical path length. In addition, the exact value of ε for the ligand can hardly be found in the literature. Consequently, it is challenging to know exactly how many ligand molecules are probed to give an accurate assessment of the coverage density of the ligands on the surface. This issue can be addressed by calculating the ratio between the absorbance of ν-CH_2_ peaks and that of ν-COO­(H^+^) peaks.

Experimentally, we firstly recorded the FTIR spectrum from a solid mixture of citric acid and CTAC at the same molar number, and the ratio between the absorbance of the ν-COOH peak and that of the ν-CH_2_ peak was found to be 0.64 (Figure S14). This ratio was calculated using the absorbance at 1710 cm^–1^ (*A*_C=O_), corresponding to the stretching of the C=O group, and the absorbance of the peak at 2920 cm^–1^ (*A*_CH_2__), corresponding to the asymmetric stretching of the CH_2_ group. Using this absorbance ratio and Equation 2, we further derived a simple equation relating the ratio of *A*_C=O_ to *A*_CH_2__ recorded from nanocrystals with different shapes to the molar ratio of surface-bound tri-citrate­(3H^+^) to CTAB/C (*c*-tri-citrate­(3H^+^)/*c*-CTA^+^), as shown in Equation 3 (see the Experimental Section):

$$A_{C=O}/A_{\mathrm{CH}2}=\left(3.2\right)\cdot \left(3/15\right)\cdot \left(c‐\mathrm{tri}‐\mathrm{citrate}\left(3H^{+}\right)/c‐{\mathrm{CTA}}^{+}\right)$$ 3

where *c*-tri-citrate­(3H^+^) or *c*-CTA^+^ referred to the total concentration (free plus surface-bound) of tri-citrate­(3H^+^) or CTA^+^ being measured. Since the free ligands were extensively removed by washing (see the Experimental Section), the measured concentration can be approximated as the concentration of the surface-bound ligand. We then calculated the ratio of *A*_C=O_ to *A*_CH_2__ for nanocrystals obtained at different stages of ligand exchange (Figure 7). At *t* = 5 min, the ratio of absorbance for 10-nm spheres (black trace), 20-nm spheres (green trace), 21-nm cubes (red trace), and 21-nm octahedra (blue trace) were 2.2, 2.1, 2.2, and 0.9, respectively, which gave molar ratios of surface-bound tri-citrate­(3H^+^) to CTAB/C at 3.3, 3.2, 3.3, and 1.4 for the samples with different shapes. The relatively low ratios confirmed the presence of a comparable amount of tri-citrate­(3H^+^) and CTAB/C on the particle surface, implying incomplete ligand exchange for all shapes within a short period of 5 min. At *t* = 30 min, the ratios of absorbance for the cubic and octahedral nanocrystals remained low at 1.9 and 1.1, respectively, corresponding to molar ratios of surface-bound tri-citrate­(3H^+^) to CTAB/C at 3.0 and 1.8. Such low values signified that most of the surface-bound CTAB/C were not replaced by tri-citrate­(3H^+^) in 30 min. In contrast, the ratio of absorbance for 10- and 20-nm Au spheres gradually increased to 4.7 and 3.9, respectively, at *t* = 30 min, corresponding to a molar ratio of surface-bound tri-citrate­(3H^+^) to CTAB/C at 7.4 and 6.1, respectively. These high ratios implied that the surface-bound CTAB/C was increasingly replaced by tri-citrate­(3H^+^) over time. As mentioned in Equation 1, the remaining small amount of CTAB/C on the surface could arise from the electrostatic trapping by tri-citrate­(3H^+^) on the Au surface. Taken together, we can conclude that only Au nanospheres exhibited an increase in the ratio of *c*-tri-citrate­(3H^+^) to *c*-CTAC, confirming that they were advantageous in enabling ligand exchange compared to their cubic and octahedral counterparts. The efficacy of ligand exchange across different Au facets descended in the order of high-index facets > {100} > {111}. This trend could suggest a stronger binding of tri-citrate­(3H^+^) to high-index facets compared to low-index ones, and this trend is supported by the results from a computational study in the next section.

**Figure 7:**
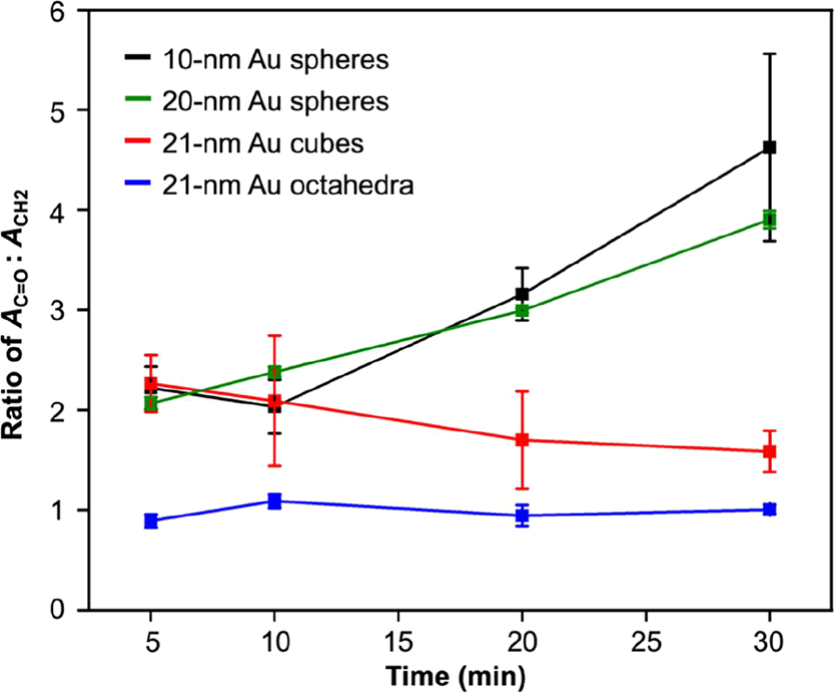
Ratios of *A*_C_=_O_ to *A*_CH_2__ in the FTIR spectra recorded from Au nanocrystals with different shapes at different stages of ligand exchange.

The incomplete ligand exchange on cubic and octahedral nanocrystals would impede their biomedical applications. To improve the outcome, we extended the exchange time from 30 min to 24 h while increasing the concentration of citric acid by fivefold. Using the optimized protocol, we also replaced the CTAB/C on Au nanorods with tri-citrate­(3H^+^) to demonstrate the universality of our method. As shown in Figure S15, the rods had an average diameter of 27 and an average length of 72 nm. It is worth noting that the nanorods assembled in an end-to-end configuration after ligand exchange. As reported in the literature,^48^ the assembly might arise from London–van der Waals attractive forces between the particles. In addition, the tips of nanorods, which were reported to be capped by low-index facets,^49^ had a lower density of tri-citrate­(3H^+^) than the side faces, which were capped by high-index facets,^49^ due to a weaker binding strength. Consequently, electrostatic repulsion between the tips was weaker than that between the side faces, allowing the attractive force to more easily overcome the repulsion and, thus, induce the tip-to-tip assembly. Such tip-to-tip assembly induced by a lower density of surface ligand on the tips when compared to the side faces has also been reported in the literature.^50^ Figure 8A shows the FTIR spectra recorded from the Au nanocrystals with various shapes after ligand exchange using the optimized protocol. All the samples showed the presence of ν-COO­(H^+^) peaks at 1200 and 1700 cm^–1^, together with the absence of ν-CH_2_ peaks around 2900 cm^–1^, indicating that their surface was covered by tri-citrate­(3H^+^) rather than CTAB/C.

**Figure 8:**
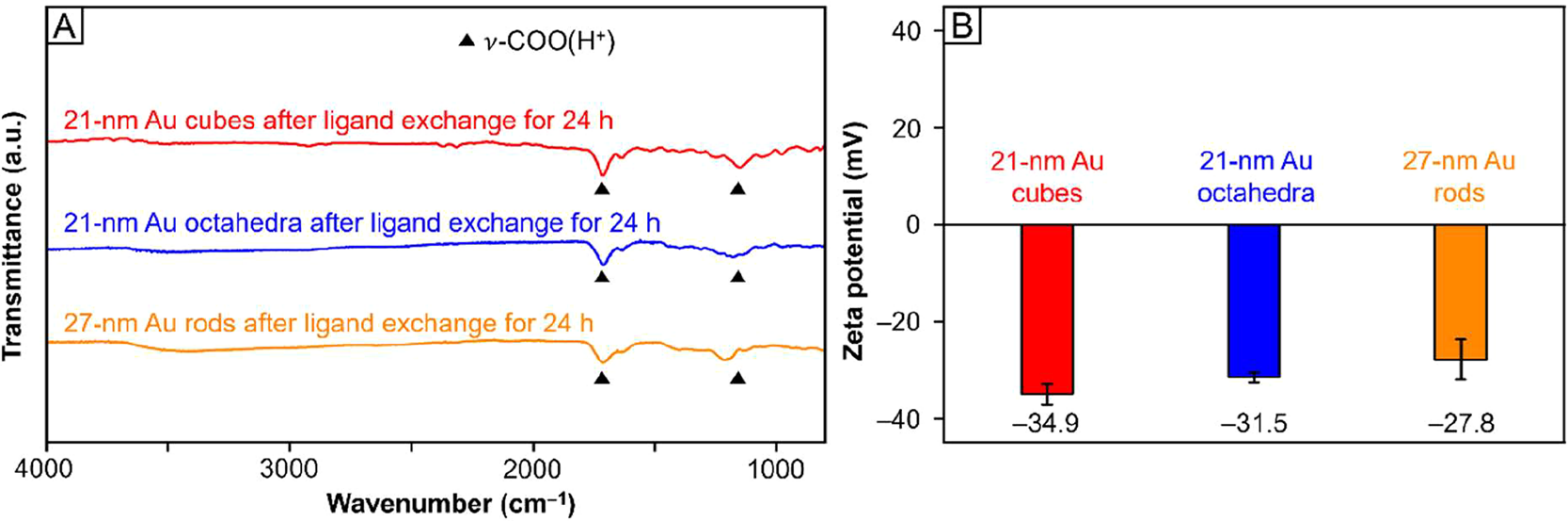
(A) FTIR spectra and (B) zeta potentials recorded from 21-nm cubes, 21-nm octahedra, and 27-nm rods after ligand exchange for 24 h with aqueous citric acid at a final concentration of 50 mM (pH = 2). The pH used for zeta potential measurements is 12.

We also measured the zeta potential of the samples (Figure 8B). All of them showed negative potentials of – 30 mV at pH = 12, which were consistent with the value measured for Au nanospheres. The consistency in zeta potential implied that the surface of the Au nanocrystals was mainly covered by tri-citrate­(3H^+^). Altogether, the FTIR and zeta potential measurements suggested that most of the CTAB/C could be replaced by increasing the time for ligand exchange and/or the concentration of citric acid. Accordingly, it can be concluded that Au nanocrystals of different shapes may require different conditions for complete ligand exchange, with nanospheres being the easiest to achieve. We also recorded the UV–vis spectra of suspensions containing different Au nanocrystals after ligand exchange (Figure S16). Overall, the LSPR peak width and position were largely preserved when replacing the CTAB/C with tri-citrate­(3H^+^), indicating that the colloidal stability of nanocrystals was not compromised upon ligand exchange.

### DFT Calculations for Determining the Binding Energies of Surface Adsorbates

To better understand the strengths of interaction between the various adsorbates and Au surfaces relevant to our experiments, we performed DFT calculations. Specifically, we studied citrate species with relevant molecular identities and CTAB/C, all with different Au facets. A previous study from our group suggested that the 10-nm Au spheres could be enclosed by a combination of {100}, {111}, and high-index facets including {211} and {331}.^51^ The technical details of DFT calculations are provided in the Experimental Section. The exchange–correlation functional used in our calculations was PBE+D3. The binding energy of each adsorbate considered here on each Au facet (BE_adsorbate_) was evaluated using Equation 4:

$${\mathrm{BE}}_{\mathrm{adsorbate}}=E_{\mathrm{ps}+\mathrm{adsorbate}}-E_{\mathrm{ps}}-E_{\mathrm{gas}}$$ 4

where *E*_ps+adsorbate_ represents the total energy of the pristine slab exposing the respective Au facet with the adsorbate at its minimum energy configuration and *E*_ps_ and *E*_gas_ denote the total energies of the respective pristine Au slab and the isolated adsorbate in the gas phase, respectively. For all surface-bound citrate species, the gas-phase reference was chosen as the fully deprotonated form of each species. In this analysis, citrate species resulting from cleavage of the O–H bond in the carboxyl group of citric acid or its derivatives demonstrated enhanced binding to the Au surface. However, when these citrate species were saturated with the appropriate number of Na^+^ counterions, the resulting citrate species bound to the Au surface more weakly than tri-citrate­(3H^+^) to the same surface. Informed by the experimental characterizations of the Au nanocrystals used in our study, we examined the interaction of adsorbed species with the following Au facets: {100}, {111}, {211}, and {331}. We then studied the adsorption of all surface-bound citrate species and the CTAB molecule on these four Au facets at the respective low coverage limit for each facet (1/32 monolayer (ML) for {100}, 1/36 ML for {111}, and 1/45 ML for {211} and {331} facets, respectively), where the adsorbate–adsorbate interactions were expected to be minimal. The most stable binding configurations for each adsorbate on these Au facets are shown in Figure 9. It should be noted that CTAB was selected as the model ligand for the calculation. As mentioned above, Br^–^ and Cl^–^ had different binding strengths to the Au surface. In fact, CTAB formed a more compact bilayer on the Au nanoparticles when compared with CTAC, implying stronger binding to the surface. In other words, CTAC was more readily replaced by tri-citrate­(3H^+^) than CTAB, an observation that was also supported by our XPS analysis. As such, using CTAB as the model offered a conservative estimate of the overall binding strength of CTAB/C to the Au surface.

**Figure 9:**
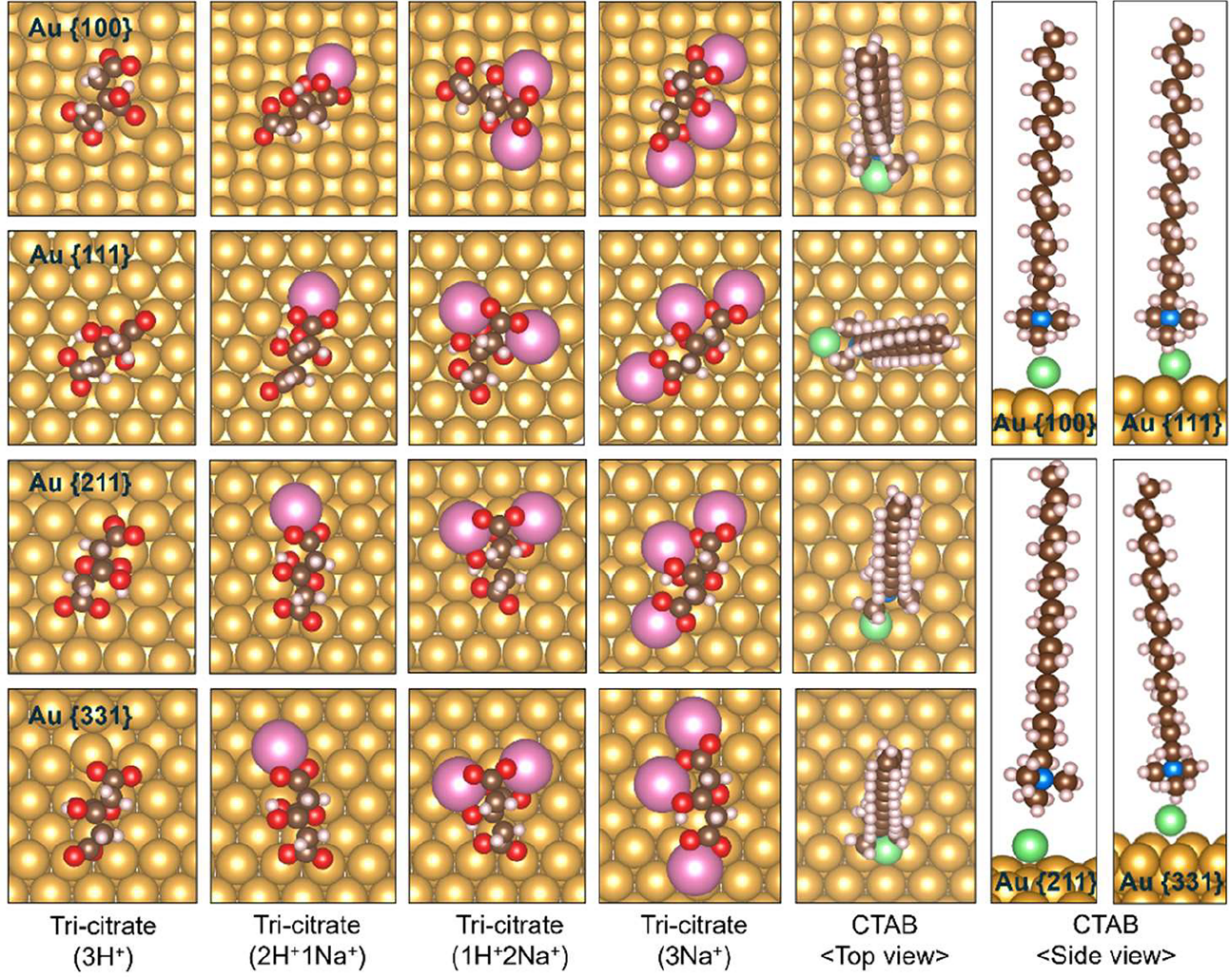
Most stable binding configurations of all surface-bound citrate species, shown in their fully deprotonated states, and CTAB on the {100}, {111}, {211}, and {331} facets at appropriate coverages. Color coding: golden, pink, green, blue, brown, white, and red spheres represent Au, Na, Br, N, C, H, and O, respectively.

Red data points in Figure 10 show that tri-citrate­(3H^+^) bound more strongly to the high-index facets (e.g., {331}, BE = −6.48 eV) compared to the low-index facets ({100}, BE = −5.80 eV; {111}, BE = −4.34 eV). In addition, a comparison of the binding energies of tri-citrate­(3H^+^) on each Au surface (red data points) with that of the CTAB molecule (purple data points) revealed that tri-citrate­(3H^+^) bound much more strongly to each Au surface than CTAB did, leading to a facile displacement of CTAB by tri-citrate­(3H^+^), which was in good agreement with our experimental findings. Furthermore, the data presented in Figure 10 also suggested that as more carboxyl moieties in citric acid were replaced by sodium-carboxylate species, the interaction of the resulting species with each Au surface became weaker. This led to a smaller driving force for the displacement of CTAB by these citrate species with the Na^+^ counterion compared to the driving force for the displacement of CTAB by tri-citrate­(3H^+^), a result that rationalized the experimental observations discussed above. Finally, the results in Figure 10 also suggested that ligand exchange effectiveness, as reflected in the thermodynamic driving force for the CTAB displacement by all surface-bound citrate species, was maximized for the more open facets (e.g., {331} and {100}) and minimized for the closest packed (111) surface. As a result, a longer time and/or a higher concentration of citric acid were needed for achieving complete ligand exchange on cubic, octahedral, and rod-like nanocrystals when compared with the spherical counterpart.

**Figure 10:**
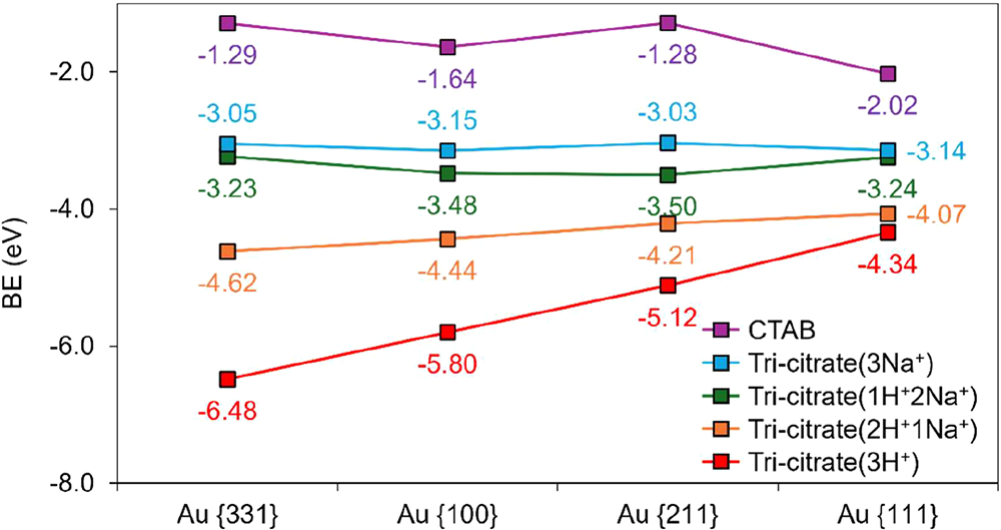
Calculated binding energies for the most stable adsorption states of each adsorbate on different Au facets. For all surface-bound citrate species, binding energies were referenced to their fully deprotonated gas-phase forms. Negative values indicate exothermic adsorption of the gas-phase species on the Au surface. Lines connecting data for each adsorbed species serve only as guides to the eye.

## Conclusions

In summary, we have demonstrated a facile method for directly replacing the toxic CTAB/C on Au nanocrystals with tri-citrate­(3H^+^). Specifically, tri-citrate­(3H^+^) rapidly replaces CTAB/C by forming stronger bonds with the Au surface. However, as the H^+^ counterion alongside the carboxylate group is replaced by Na^+^, which, according to our experimental and computational studies, destabilizes the binding between carboxylate group and the Au surface, the ligand exchange became slow and practically unsuccessful. Quantitative FTIR analyses integrated with DFT calculations further indicate that tri-citrate­(3H^+^) binds more strongly to the high-index facets on Au nanospheres when compared with the low-index facets on cubic, octahedral, or rod-like Au nanocrystals. The stronger binding results in more efficient ligand exchange on spherical nanocrystals when compared to other shapes, allowing the ligand exchange to complete within a shorter time. While the size and shape of nanocrystals can influence the efficiency of ligand exchange, it is impossible to exhaustively examine all combinations in one study or report. In general, one can increase the incubation time and/or the concentration of citric acid to achieve complete ligand exchange on low-index facets than the high-index ones. The findings are expected to be extendable to essentially all types of Au nanocrystals with different shapes that have found widespread use in biomedical applications. Notable examples may include star-like or spherical nanocrystals enclosed by high-index facets;^52^ octahedra, icosahedra, decahedra, and triangular plates covered by {111} facets;^53^ cubes and cubic cages covered by {100} facets;^54^ and nanorods or nanowires covered by {100} and/or {110} side faces.^55^ Our insights into the binding mechanism of citrate species with the Au surface also offer guidelines for achieving direct ligand exchange without eliciting particle agglomeration. The experimental approach should be extensible to the other pairs of ligands and different metal surfaces.

## Experimental Section

### Chemicals and Materials

Gold­(III) chloride trihydrate (HAuCl_4_·3H_2_O, ≥99.9%), sodium borohydride (NaBH_4_, 98%), ascorbic acid (AA, ≥99.0%), cetyltrimethylammonium bromide (CTAB, ≥99.0%), cetyltrimethylammonium chloride (CTAC, 25% in water), citric acid (≥99.0%), trisodium citrate (≥99.0%), HCl (37%), and NaOH (≥99.0%) were all obtained from Sigma-Aldrich and used as received. Deionized water with a resistivity of 18.2 MΩ·cm at room temperature was used throughout the experiments.

### Synthesis of 10-nm Au Spheres Capped by CTAC/B

We followed a published protocol to synthesize CTAC/B-capped Au nanospheres 10 nm in diameter. In brief, Au clusters were first prepared by mixing 5 mL of aqueous CTAB (200 mM) and 5 mL of aqueous HAuCl_4_ (0.5 mM) in a 20 mL glass vial, followed by the introduction of freshly prepared 0.6 mL of aqueous NaBH_4_ (10 mM) in one shot at 27 °C. The mixture was placed on an orbital shaker at a speed of 270 rpm for 2 min and then kept undisturbed at 27 °C for at least 3 h to allow the NaBH_4_ to completely decompose. Meanwhile, 2 mL of aqueous CTAC (200 mM) and 2 mL of aqueous HAuCl_4_ (0.5 mM) were mixed in a separate 20 mL glass vial, followed by a one-shot injection of 1.5 mL of aqueous AA (100 mM) under magnetic stirring at a speed of 600 rpm and at 27 °C. Then, 50 μL of the aqueous suspension of Au clusters was introduced. The reaction was allowed to proceed at 27 °C for 15 min. The solid products were collected by centrifugation at 14500 rpm for 30 min and washed once with water. After the removal of supernatant, the particles were redispersed in 1 mL of aqueous CTAC (20 mM).

### Exchange of CTAC/B with Tri-citrate­(3H^+^) on the Surface of 10-nm Au Spheres

In a standard protocol, a solution was first prepared by mixing 130 μL of aqueous citric acid (200 mM), 200 μL of aqueous HCl (100 mM), and 2 mL of water in a 20 mL glass vial at a speed of 600 rpm at 27 °C. The pH of this solution was confirmed to be 2 by measuring with a pH meter. Subsequently, 200 μL of the aqueous suspension of Au nanospheres (6.22 × 10^10^ particles per mL in 20 mM aqueous CTAC) was introduced in one shot. The ligand exchange was allowed to proceed at 27 °C for 30 min. The solid products were collected by centrifugation at 14500 rpm for 20 min, washed with water, and then redispersed in 500 μL of 50 mM aqueous citric acid (pH = 2) for storage or in PBS solution with different dilution factors to test the colloidal stability under different ionic strengths (see the main text for details).

For ligand exchange on 20- and 60-nm spheres, 21-nm cubes, 21-nm octahedra, and 27-nm rods, Au nanocrystals with different shapes were first prepared by modifying the protocols reported in previous publications.^56–59^ As for ligand exchange, 200 μL of the aqueous suspension of spherical, cubic, octahedral, and rod-like Au nanocrystals (at concentrations of 1.25 × 10^12^, 1.36 × 10^12^, and 3.47 × 10^12^ particles per mL, respectively, in 20 mM aqueous CTAC) was used, while all other parameters were kept the same as those in the case of 10-nm nanospheres. See Figure S17 and Table S1 for the size distributions of the different Au nanocrystals.

For ligand exchange at different pH, aqueous HCl or NaOH was added prior to the introduction of Au nanospheres (see Table 2 for details). All other parameters were kept the same as those in the case of pH = 2.

**Table 2:** HCl or NaOH Added to the Reaction Mixture for Ligand Exchange at Different pHs

pH value of the mixture	dominating citrate species in the solution	concentration of HCl or NaOH	volume added (μL)
2	citric acid	HCl (0.01 M)	200
4	mono-citrate	NaOH (0.01 M)	50
5.8	bi-citrate	NaOH (0.01 M)	95
8	tri-citrate	NaOH (0.01 M)	115

### ATR-FTIR Measurement

The sample of nanocrystals for ATR-FTIR measurement was washed multiple times with water prior to the measurements to remove free ligands from the sample. During each washing step, the nanocrystals were redispersed in water, followed by centrifugation and removal of the supernatant (together with the free ligand). The washing procedure was repeated until the particles could not be dispersed in water under sonication due to irreversible agglomeration (Figure S18). According to the Langmuir model, agglomeration indicated an inadequate surface coverage of the ligand caused by the depletion of free ligand in the suspension medium. The agglomerated particles were collected and directly placed on the ATR crystal for FTIR measurements. In addition, the mass of Au nanocrystals with different shapes used for measurement was kept similar for a fair comparison, as confirmed by ICP-MS measurements.

### Characterizations

TEM images were taken using a Hitachi HT7700 microscope operated at 120 kV. HAADF STEM imaging was conducted using an aberration-corrected Hitachi HD-2700 200 kV STEM. The metal contents of the samples were analyzed using an ICP-MS (NexION 300Q, PerkinElmer). All UV–vis spectra were recorded on a Cary 60 spectrometer (Agilent Technologies). All FTIR spectra were recorded on a Varian 640 IR spectrometer (Agilent Technologies). All XPS spectra were recorded on a K-Alpha spectrometer with an Al Kα source (Thermo Fisher). Zeta potential measurements were determined using a Zetasizer Nano ZS (Malvern Instruments, Worcestershire, UK).

### Calculation of the Number of CTAB Molecules on the 10-nm Au Spheres

Each CTAB molecule contains one Br^–^. Based on the molar ratio of Br^–^ ions to all Au atoms obtained from ICP-MS analysis, the coverage density of CTAB was calculated according to the following equation:

$$\frac{{\mathrm{Br}}^{-}}{\mathrm{total}\;\mathrm{Au}}=\left[10\;\mathrm{nm}\;\mathrm{Au}\;\mathrm{spheres}\;\mathrm{surface}\;\mathrm{area}\;\left({\mathrm{nm}}^{2}\right)\times \mathrm{CTAB}\;\mathrm{coverage}\;\mathrm{density}\;\left(\frac{\mathrm{molecules}}{{\mathrm{nm}}^{2}}\right)\right]/\left[\mathrm{number}\;\mathrm{of}\;\mathrm{Au}\;\mathrm{atoms}\;\mathrm{per}\;\mathrm{sphere}\right]=\frac{4\pi \cdot r^{2}\cdot \phi_{\mathrm{CTAB}}}{4\cdot \left(\frac{\frac{4}{3}\pi \cdot r^{3}}{a^{3}}\right)}=3\cdot \frac{a^{3}\cdot \phi_{\mathrm{CTAB}}}{4r}$$

Rearranging the equation gives

$$\phi_{\mathrm{CTAB}}=\frac{4}{3}\cdot \frac{r}{a^{3}}\cdot \frac{{\mathrm{Br}}^{-}}{\mathrm{total}\;\mathrm{Au}}$$

where *r* is 5 nm, *a* is the lattice constant of Au (0.408 nm), and ϕ_CTAB_ is the coverage density of CTAB molecules (nm^–2^). For each 10-nm Au sphere, the surface area is 62.8 nm^2^; hence, the number of CTAB molecules on each 10-nm Au sphere equals ϕ_CTAB_ × 62.8.

### Derivation of eq 3

Since each CTAC molecule contains 15 CH_2_ groups and each tri-citrate­(3H^+^) molecule contains three C=O groups, the ratio of *A*-C=O to *A*-CH_2_ can be derived based on Equation 2:

$$\frac{A‐C=O}{A‐{\mathrm{CH}}_{2}}=\frac{\varepsilon ‐C=O\cdot 3\cdot c‐\mathrm{tri}‐\mathrm{citrate}(3H^{+})\cdot l}{\varepsilon ‐{\mathrm{CH}}_{2}\cdot 15\cdot c‐\mathrm{CTAC}\cdot l}$$ 5

To calculate *c*-tri-citrate­(3H^+^)/*c*-CTA^+^, one has to determine the ratio of ε-C=O to ε-CH_2_. In the current study, this ratio was calculated according to the ratio of *A*-C=O to *A*-CH_2_ obtained in Figure S14 (0.64), as shown in Equation S2:

$$0.64=\frac{\varepsilon ‐C=O\cdot 3}{\varepsilon ‐CH_{2}\cdot 15}$$ 6

where *c*-CTAC canceled out *c*-tri-citrate­(3H^+^) since the molar numbers were the same. The *l*-values also canceled out in the numerator and denominator because of the use of a homogeneous mixture of solid CTAC and citric acid. Consequently, the ratio of ε-C=O to ε-CH_2_ was calculated to be 3.2. Subsequently, Equation 3 was derived by substituting the ratio of ε-C=O to ε-CH_2_ into Equation S1:

$$\frac{A‐C=O}{A‐{\mathrm{CH}}_{2}}=(3.2)\cdot \left(\frac{3}{15}\right)\cdot \frac{c‐\mathrm{tri}‐\mathrm{citrate}(3H^{+})}{c‐\mathrm{CTAC}}$$ 7

Again, the *l*-values were canceled out in the numerator and denominator.

### Computational Details

Periodic DFT calculations were performed using the Vienna ab initio software package (VASP).^60,61^ The Perdew–Burke–Ernzerhof (PBE) exchange–correlation functional within the generalized gradient approximation (GGA) was employed.^62^ The long-range dispersive interactions, which are poorly described by PBE alone, were accounted for using Grimme’s D3 method (PBE + D3).^63,64^ The projector augmented wave (PAW) method using a plane-wave basis set was implemented to describe the ion–electron interactions.^65^ The Kohn–Sham electron eigenfunctions were expanded in plane-wave basis sets with an energy cutoff of 400 eV. Structures were optimized until the self-consistent energies and residual forces on all the constituent atoms became smaller than 10^–4^ eV and 0.02 eV/Å, respectively. Convergence with respect to all computational parameters was checked. To determine the equilibrium geometries and total energies, the first Brillouin zone integration of each unit cell was sampled with a (3 × 3 × 1) Monkhorst–Pack mesh of k-points.^66^ Given the large number of Au atoms per layer in the Au slabs, surface adsorption calculations were performed on three-layer slabs infinitely repeated in a supercell geometry, with the bottom two atomic layers of the metal slab fixed. Any pair of successive slabs in the *z*-direction was separated by a vacuum layer of varying thickness, which allowed for at least 15Å of separation to prevent the interaction between periodic images. Dipole correction was applied to all slab calculations.^67,68^ To approximately match the number of atoms in each unit cell, cubic {100} and octahedral {111} surfaces were infinitely repeated in (4 × 4) and (6 × 6) supercell geometry, including 32 and 36 surface atoms per unit cell, respectively. For the {211} and {331} surfaces, a (5 × 3) supercell with 45 surface atoms was utilized. The calculated lattice constant for Au is 4.100 Å, which is in good agreement with the experimental value (4.078 Å).

## Supplementary Material



## Notes

Speciation diagram of citric acid (Figure S1); schematic showing the binding of different citrate species to a Au atom (Figure S2); calculated wavenumbers of the C=O bond in different citrate species when binding to different types of Au planes (Figure S3); zeta potentials (Figure S4); XPS spectra (Figure S5); schematic showing the details of ligand exchange (Figure S6); UV–vis spectra of tri-citrate­(3H^+^)-capped 10-nm Au spheres (Figure S7); ligand exchange on different size of Au nanospheres (Figure S8); FTIR spectra of ligand exchange at different pH using KOH (Figure S9); UV–vis spectra of dilution test (Figure S10); Langmuir curves (Figure S11); UV–vis spectra of ligand exchange at different pH (Figure S12); TEM images of Au nanocrystals (Figure S13); FTIR spectra of a mixture of CTAC and citric acid (Figure S14); TEM images of Au nanorods (Figure S15); UV–vis spectra of different Au nanocrystals (Figure S16); size distribution of different Au nanocrystals (Figure S17 and Table S1); schematic showing the preparation of FTIR samples (Figure S18) (PDF)
