# Supplementary material for: Cetyltrimethylammonium Bromide/Chloride on Gold Nanocrystals Can Be Directly Replaced with Tri-Citrate
Source: ACS Nano. 2025 Dec 3;19(49):41669–83. doi: 10.1021/acsnano.5c13995 (PMC12713779; doi:10.1021/acsnano.5c13995)
Supplement: Supplementary file 1 [file nn5c13995_si_001.pdf]

Supporting Information

The Cetyltrimethylammonium Bromide/Chloride on Gold Nanocrystals Can Be Directly Replaced with Tri-Citrate

Kei Kwan Li,† Ho Chang Song,‡ Lang Xu,‡ Yong Ding,§ Manos Mavrikakis,‡,* and Younan Xia†,¶,*

†School of Chemistry and Biochemistry, Georgia Institute of Technology, Atlanta, Georgia 30332, United States

‡Department of Chemical and Biological Engineering, University of Wisconsin–Madison, Madison, Wisconsin, 53706, United States

§School of Materials Science and Engineering, Georgia Institute of Technology, Atlanta, Georgia 30332, United States

¶The Wallace H. Coulter Department of Biomedical Engineering, Georgia Institute of Technology and Emory University, Atlanta, Georgia 30332, United States

*Corresponding authors. E-mails: emavrikakis@wisc.edu (for the computational study) and younan.xia@bme.gatech.edu (for the experimental study)

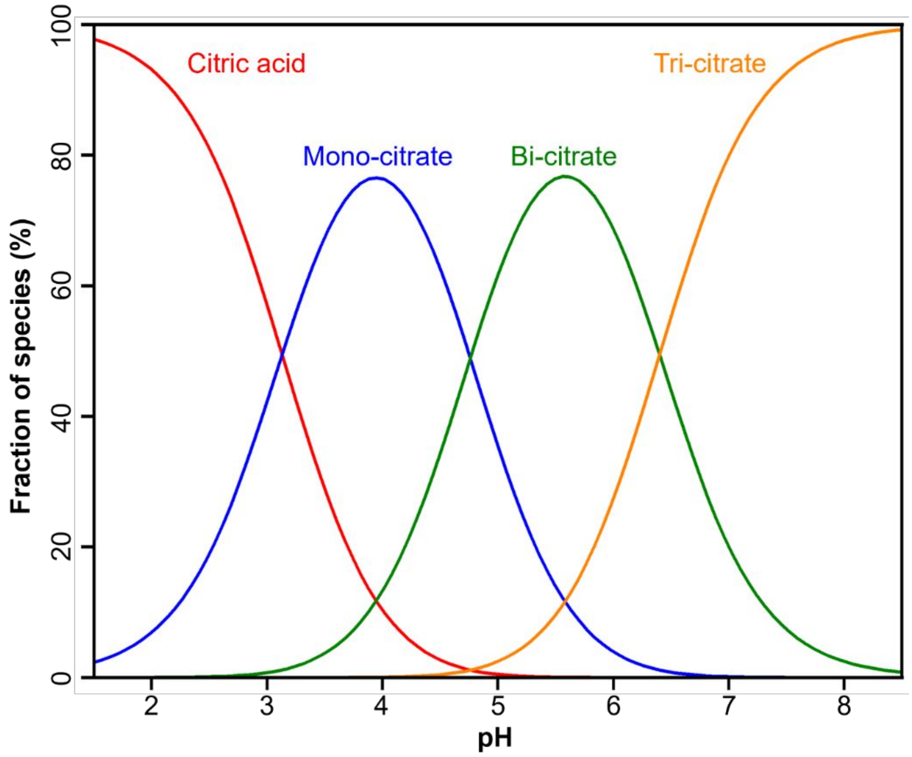

Figure S1. The speciation diagram of citric acid as a function of pH, where an increase in pH leads to the progressive deprotonation of citric acid, generating mono-, bi-, and tri-citrate. The pKa values used to generate the diagram include: $pKa_1 = 3.13$, $pKa_2 = 4.76$, $pKa_3 = 4.76$, and $pKa_4 = 6.40$.

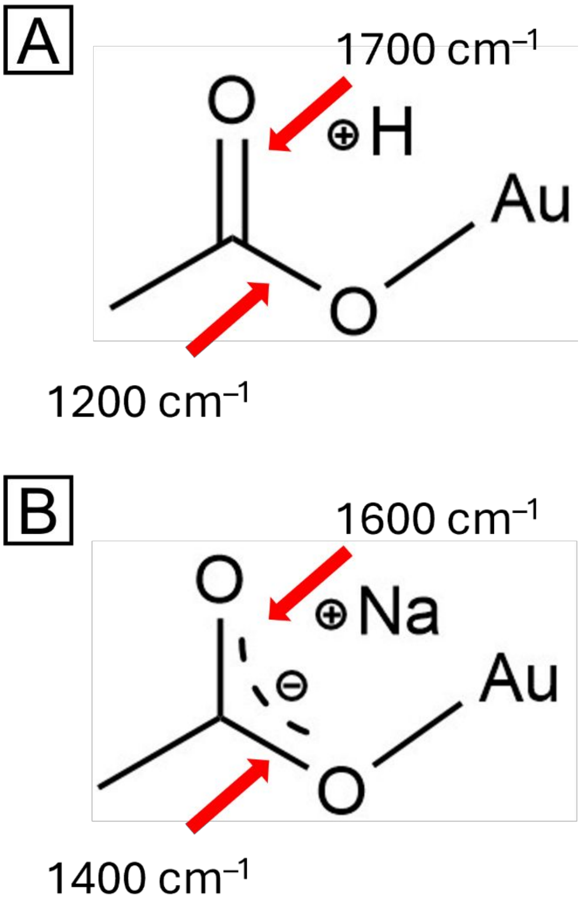

Figure S2. Schematic showing the binding of carboxyl group to a Au atom in the presence (A) H^+ and (B) Na^+ counterions, respectively, together with the corresponding wavenumbers of the two vibrational peaks.

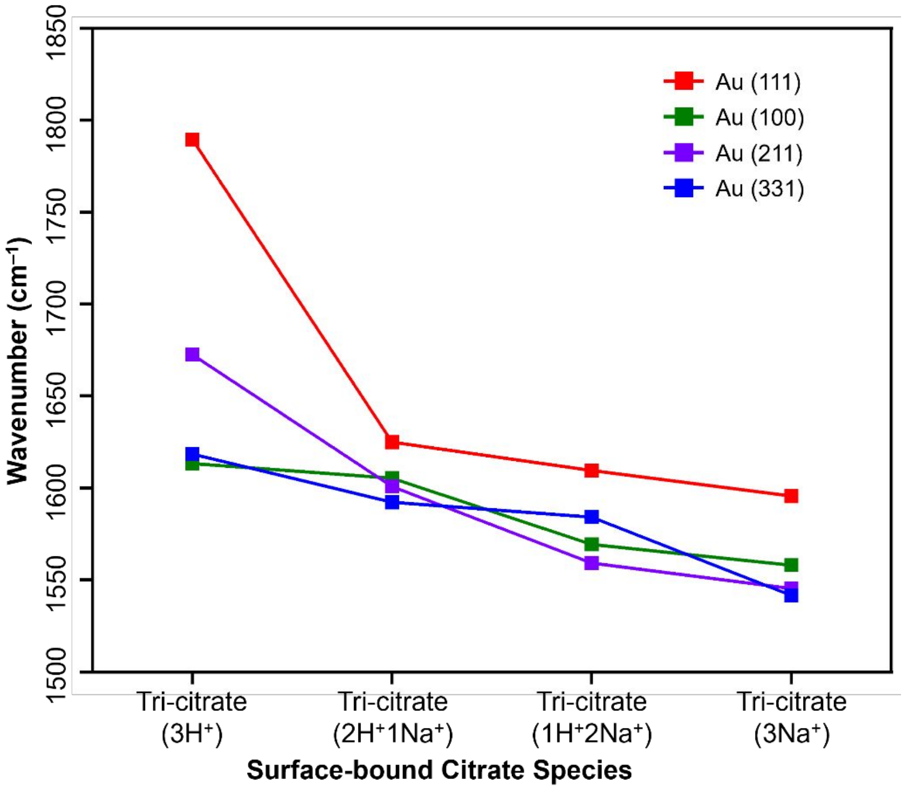

Figure S3. Calculated wavenumbers of the C=O bond in different citrate species when binding to different types of Au planes. The result gave a qualitative explanation on the red-shift of C=O stretching when the counter ions of surface-bound tri-citrate were switched from H^+ to Na^+. However, the number has no quantitative meaning and cannot be used to match the experimental data obtained in FTIR measurements.

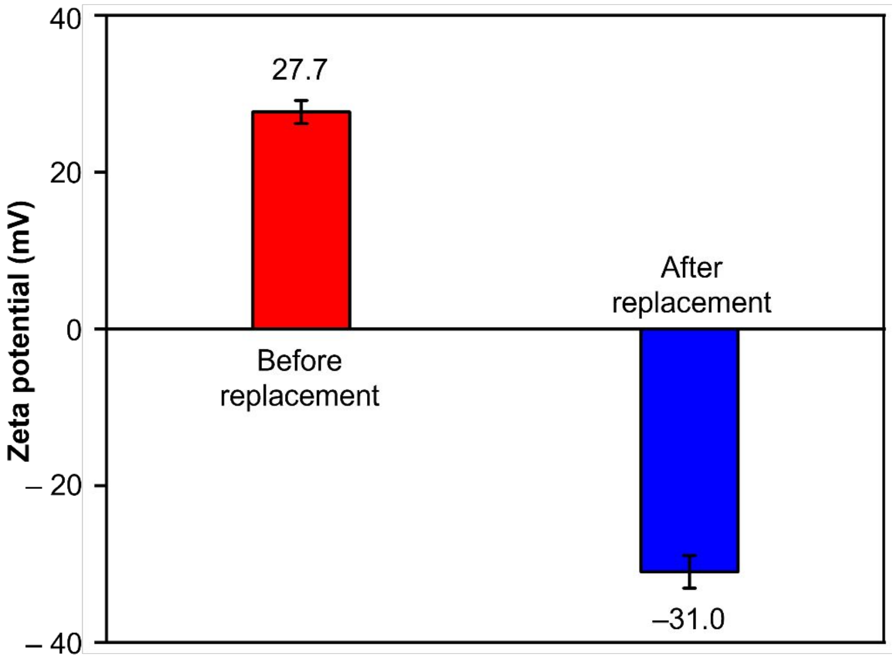

Figure S4. Zeta potentials of the 10-nm Au spheres before (red) and after (blue) ligand exchange for 30 min with aqueous citric acid at a final concentration of 10 mM (pH = 2). The pH used for zeta potential measurements was 12 to convert the tri-citrate(3H^+) to tri-citrate(3Na^+) thus matching the value of zeta potential in references (see main text for details). Unless notified, all the Au nanospheres used in this work had an average diameter of 10 nm.

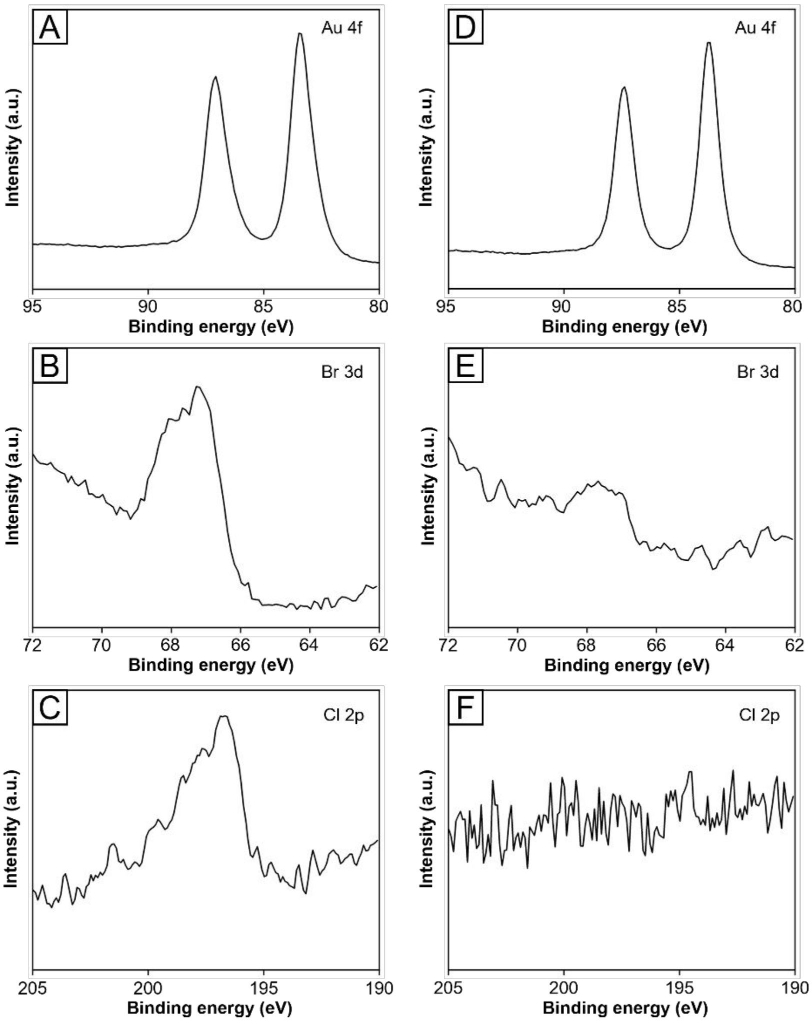

Figure S5. XPS spectra (Au 4f, Br 3d, and Cl 2p) of the 10-nm Au spheres (A-C) before and (D-F) after ligand exchange for 30 min with aqueous citric acid at a final concentration of 10 mM (pH = 2).

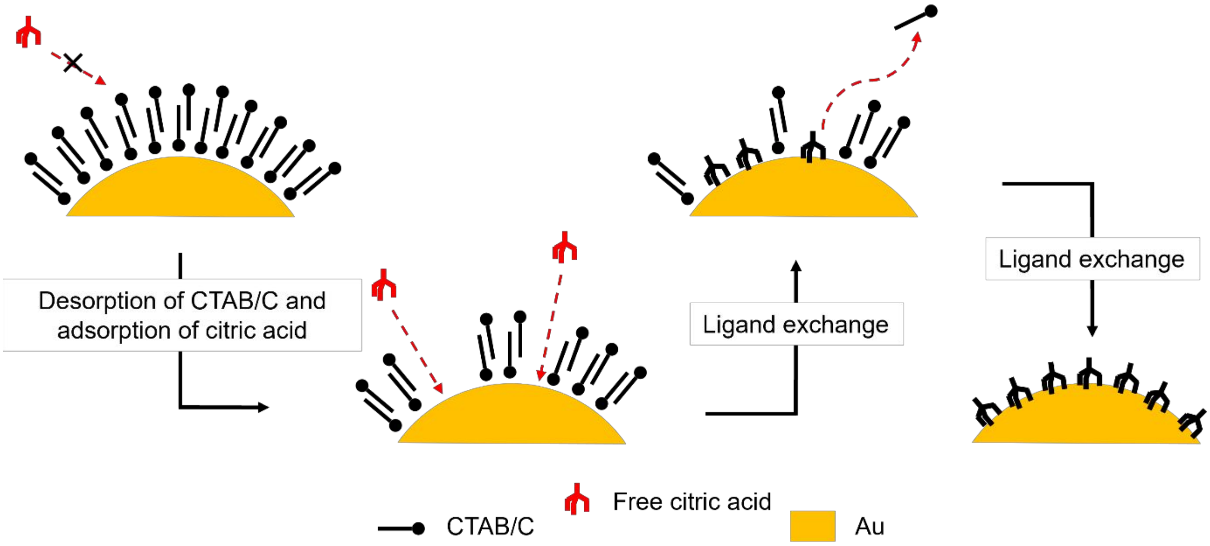

Figure S6. Schematic showing the ligand exchange between surface-bound CTAB/C and citric acid on the surface of a Au nanosphere. Upon adsorption onto the Au surface, citric acid evolves into tri-citrate with three protons as the counterions ($3H^+$-tri-citrate).

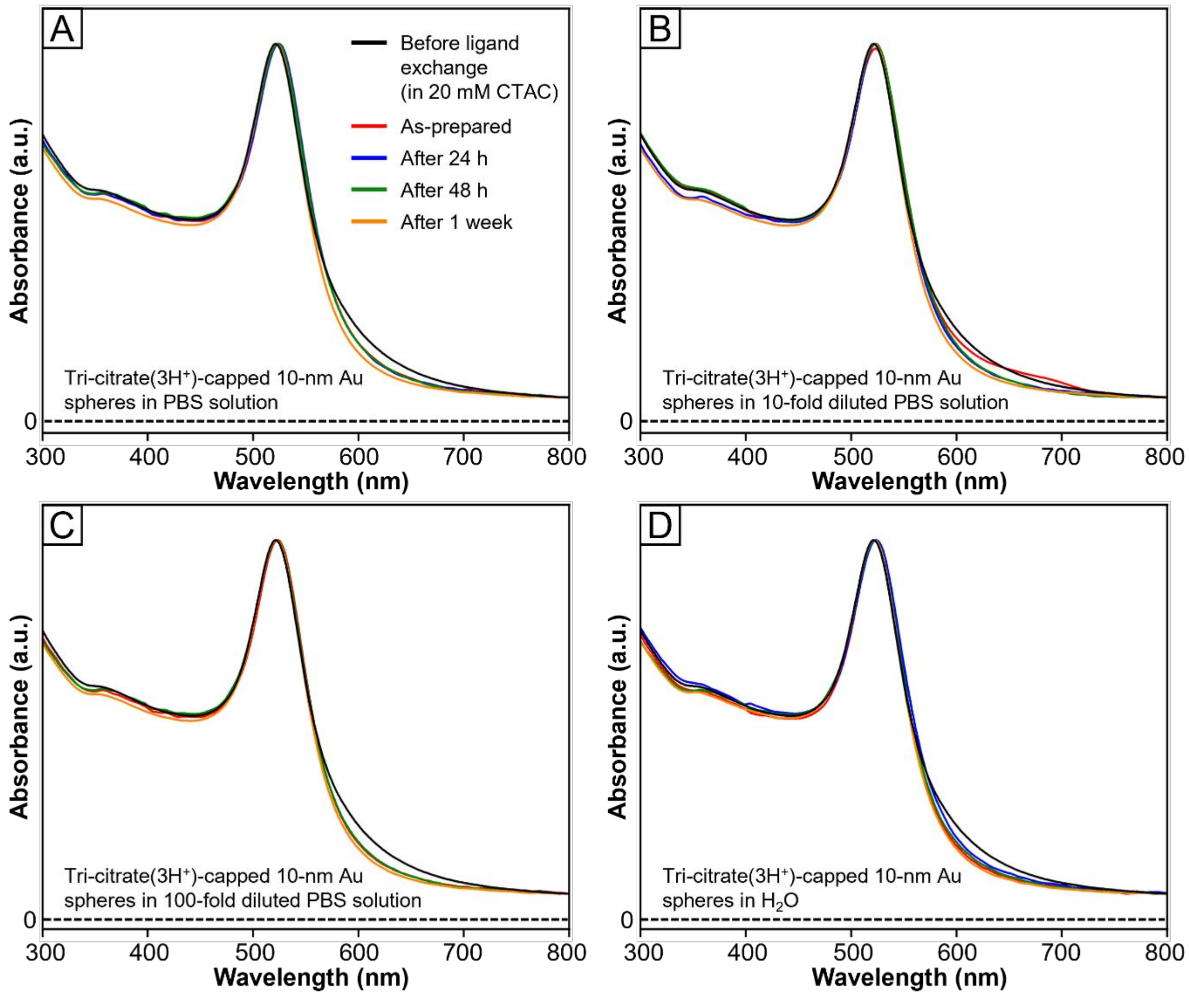

Figure S7. UV-vis spectra recorded from aqueous suspensions of tri-citrate($3H^+$)-capped 10-nm Au spheres in (A) PBS solution; (B) 10-fold diluted PBD solution; (C) 100-fold diluted PBS solution; (D) water over a period of one week. UV-vis spectra recorded from aqueous suspensions of CTAB/C-capped 10-nm Au spheres in 20 mM aqueous CTAC (black traces) were added to each figure for comparison of the colloidal stability. Note that the legend only refers to the state of the surface of the nanospheres before being dispersed in PBS. Due to the change in pH, the citrate species is expected to change from tri-citrate($3H^+$) to tri-citrate($3Na^+$) at pH = 7.

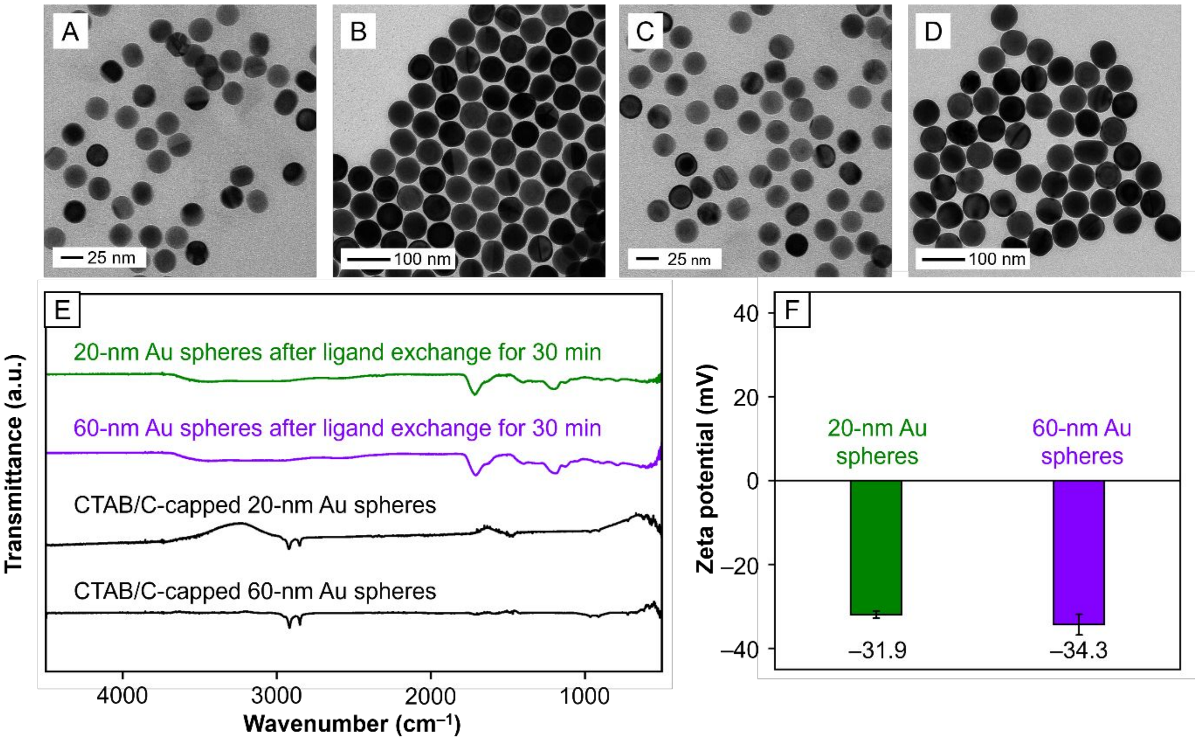

Figure S8. (A, B) TEM images of (A) 20-nm and (B) 60-nm Au spheres before ligand exchange. (C, D) TEM images of (C) 20-nm and (D) 60-nm Au spheres after ligand exchange for 30 min with aqueous citric acid at a final concentration of 10 mM (pH = 2). (E) FTIR spectra and (F) zeta potentials recorded from the 20- and 60-nm Au spheres after ligand exchange. The pH used for zeta potential measurements was 12. The pH used for zeta potential measurements was 12 to convert the tri-citrate($3H^+$) to tri-citrate($3Na^+$) thus matching the value of zeta potential in references (see main text for details).

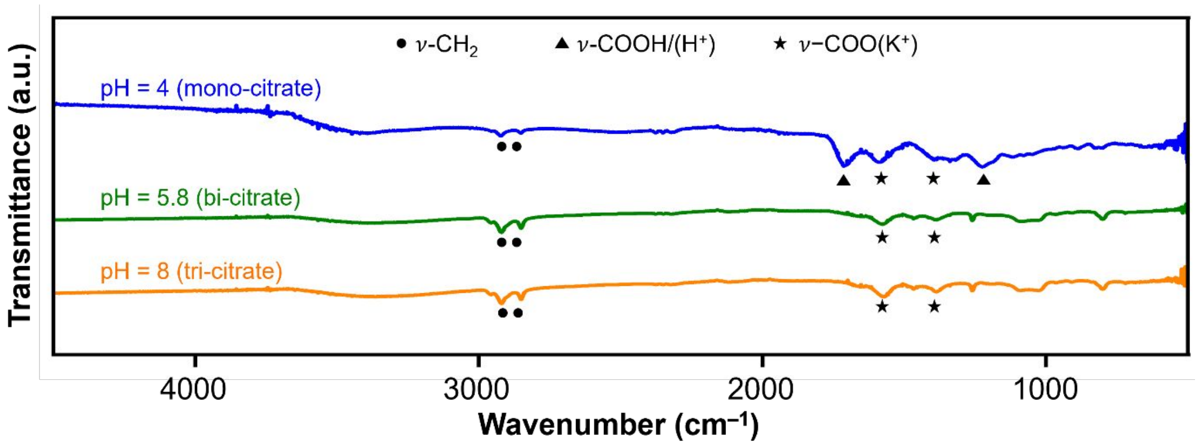

Figure S9. FTIR spectra recorded from the 10-nm Au spheres obtained after ligand exchange at different pH for 30 min using KOH instead of NaOH. The dominant citrate species in the solution at the corresponding pH is indicated in the bracket.

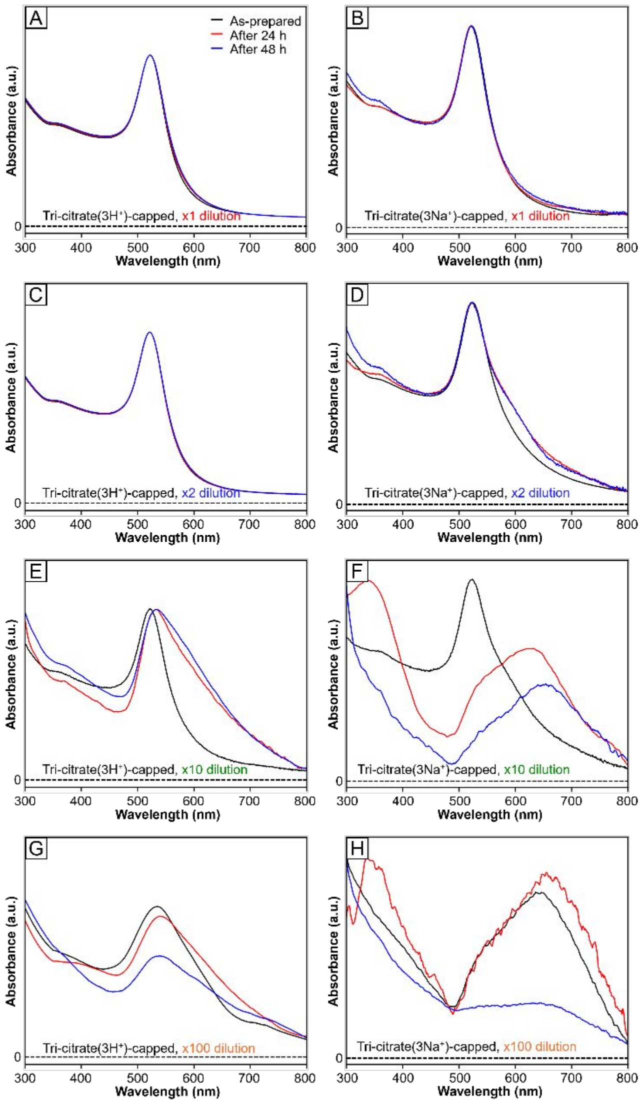

Figure S10. UV-vis spectra recorded from the aqueous suspension of the 10-nm Au spheres capped by tri-citrate($3H^+$) or tri-citrate($3Na^+$) upon dilution by different factors over 48 h: (A, B) tri-citrate($3H^+$) and tri-citrate($3Na^+$) upon x1 dilution; (C, D) tri-citrate($3H^+$) and tri-citrate($3Na^+$) upon x2 dilution; (E, F) tri-citrate($3H^+$) and tri-citrate($3Na^+$) upon x10 dilution; (G, H) tri-citrate($3H^+$) and tri-citrate($3Na^+$) upon x100 dilution. Black: sample as prepared; red: after 24 h; blue: after 48 h.

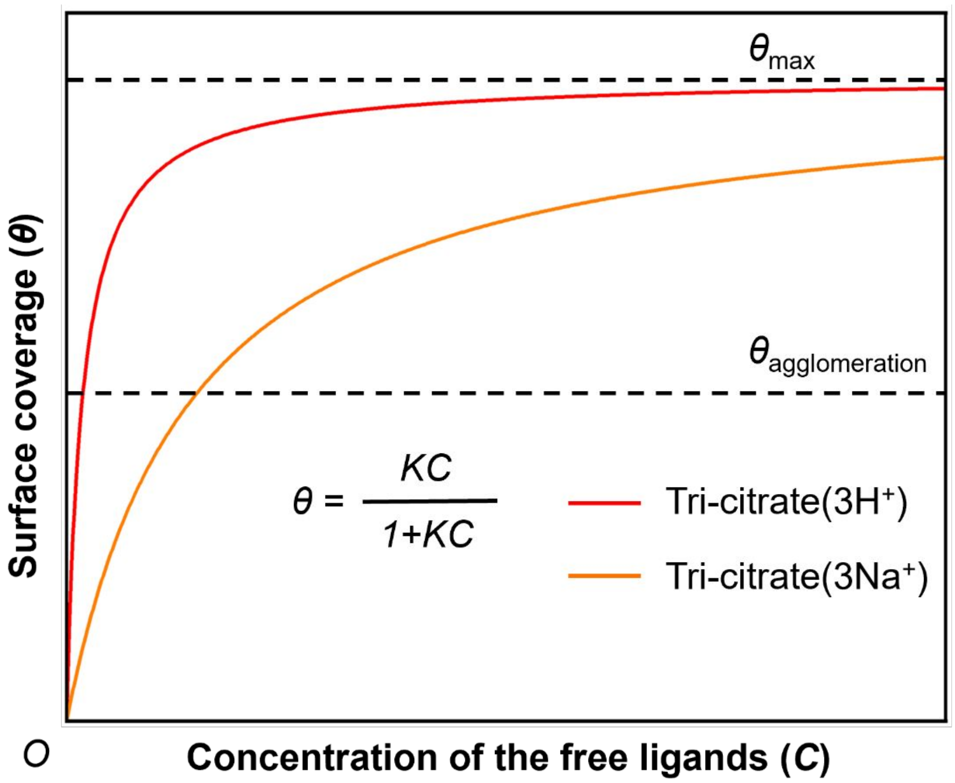

Figure S11. Langmuir curves proposed for describing the adsorption of tri-citrate($3H^+$) and tri-citrate($3Na^+$) on the surface of Au nanocrystals.

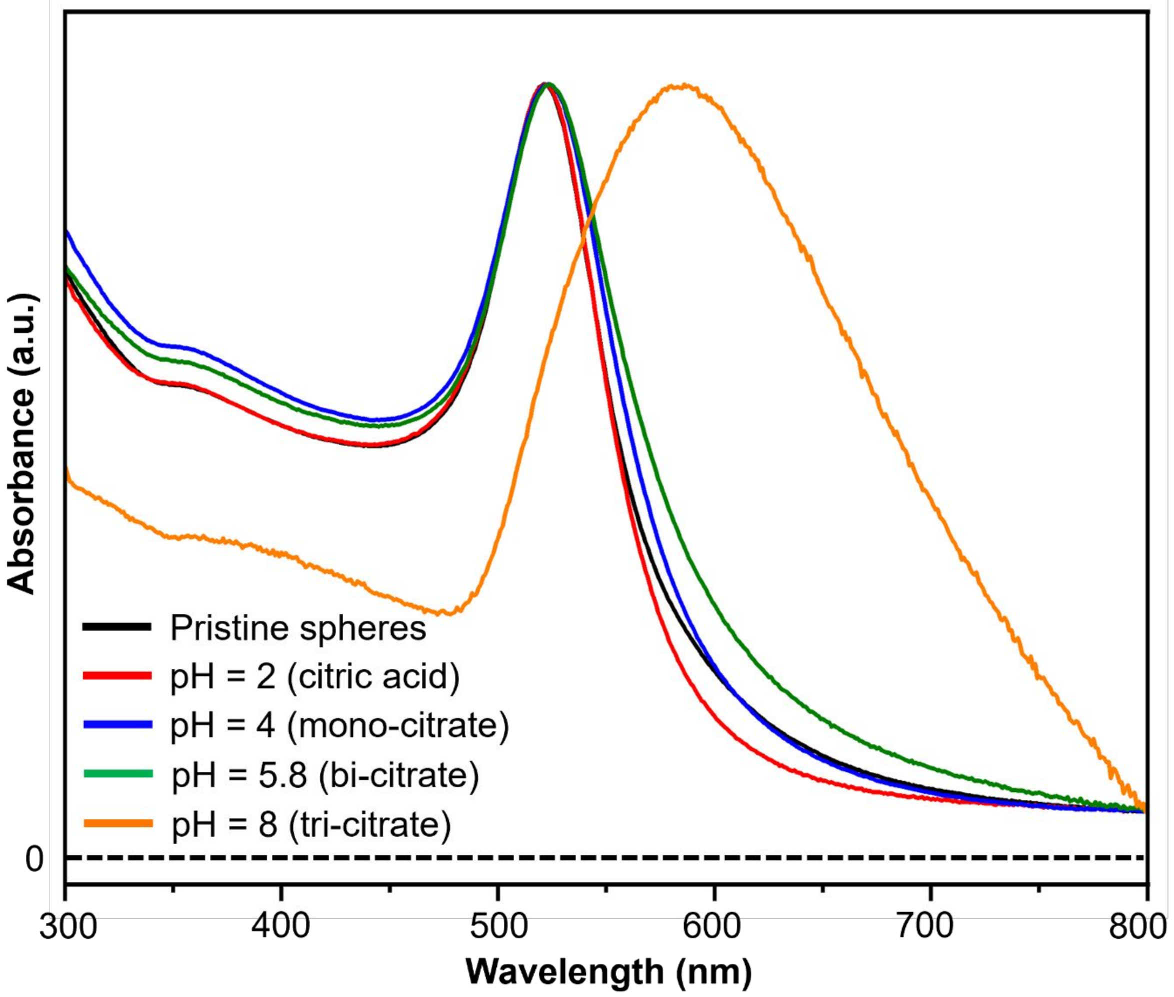

Figure S12. UV-vis spectra recorded from aqueous suspensions of the CTAB/C-capped 10-nm Au spheres in 20 mM aqueous CTAC (black) before and after direct ligand exchange at different pH for 30 min. The dominant citrate species in the solution at the corresponding pH is indicated in the bracket.

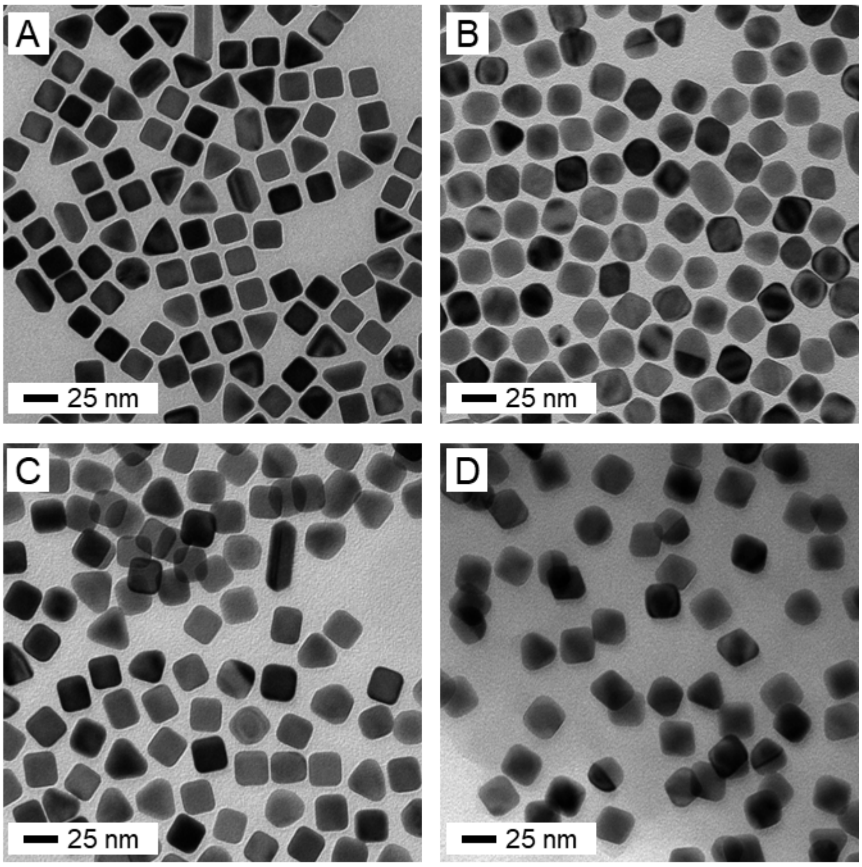

Figure S13. TEM images of the 21-nm cubes, 21-nm octahedra, and 20-nm spheres before or after the attempted ligand exchange for 30 min with aqueous citric acid at a final concentration of 10 mM (pH = 2): (A) CTAB/C-capped cubic nanocrystals before ligand exchange, (B) CTAB/C-capped octahedral nanocrystals before ligand exchange, (C) cubic nanocrystals after ligand exchange, and (D) octahedral nanocrystals after ligand exchange,

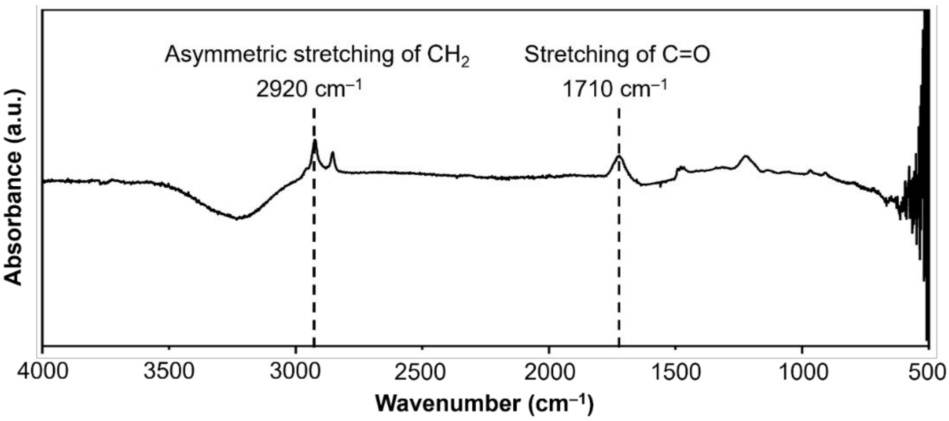

Figure S14. FTIR spectra recorded from a solid mixture of citric acid and CTAC at a molar ratio of 1:1.

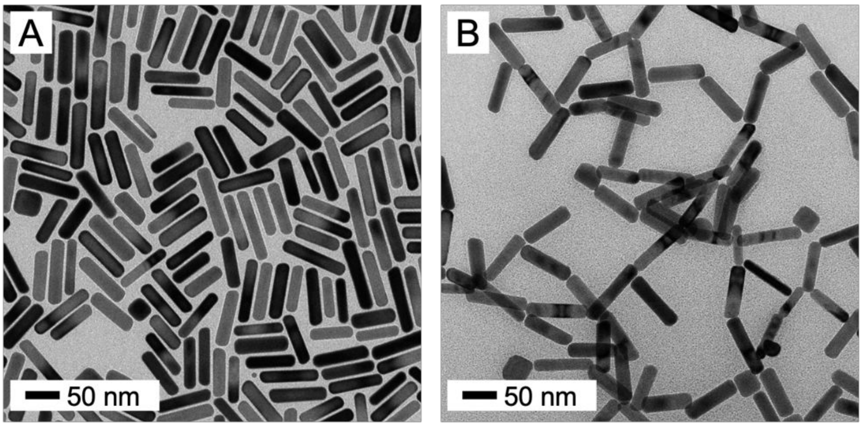

Figure S15. TEM images of the 27-nm rods (A) before and (B) after the ligand exchange for 24 h with aqueous citric acid at a final concentration of 50 mM (pH = 2). The samples for TEM imaging were prepared by dispersing the tri-citrate(3H$^+$)-capped Au nanorods into water and then drying on a TEM grid made by Cu.

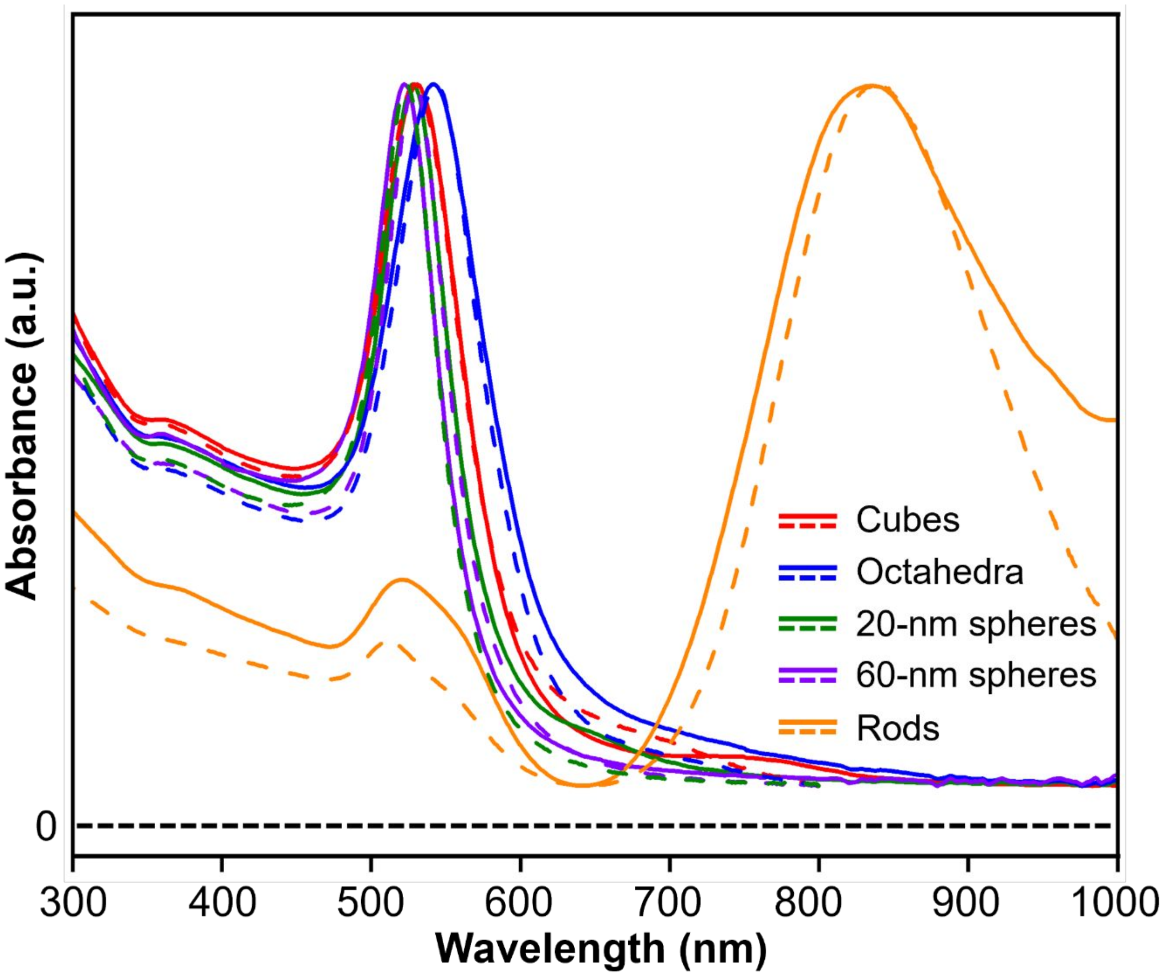

Figure S16. UV-vis spectra recorded from aqueous suspensions of Au nanocrystals with different shapes before and after ligand exchange. See the main text for a detailed description of the ligand exchange conditions. Dotted and solid lines correspond to sample before and after ligand exchange, respectively.

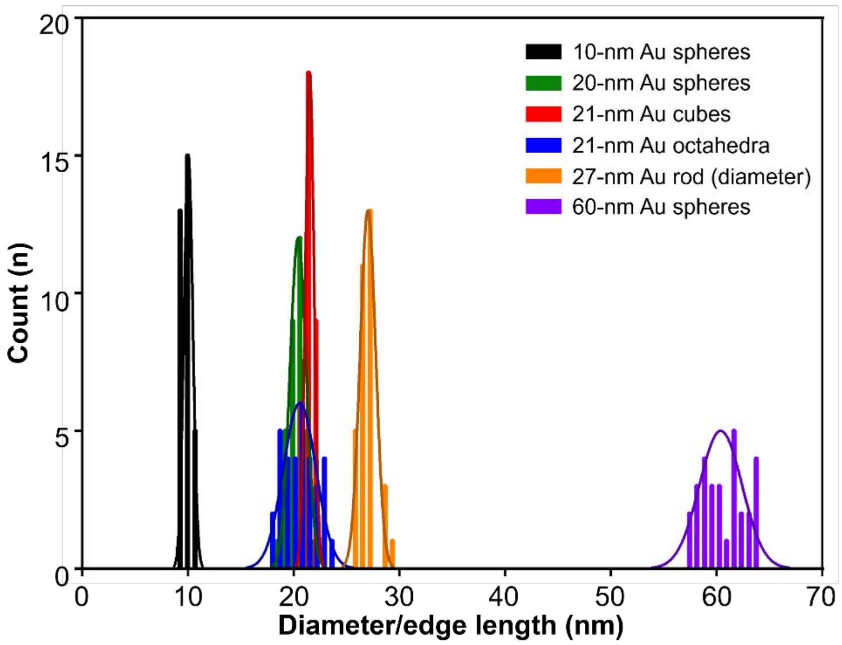

Figure S17. Distribution of diameter/edge length of Au nanocrystals with different shapes used for ligand exchange. The dimensions were measured by counting the diameter and edge length of 50 particles in the TEM images.

Table S1. Summary of diameter/edge length distribution of the Au nanocrystals with different shapes.

Shapes	Distribution of diameter/edge length
10-nm spheres	10.03 ± 0.42 nm
20-nm spheres	20.41 ± 0.77 nm
21-nm cubes	21.50 ± 0.40 nm
21-nm octahedra	21.55 ± 1.54 nm
27-nm rod (diameter)	27.01 ± 0.74 nm
60-nm spheres	60.38 ± 2.01 nm

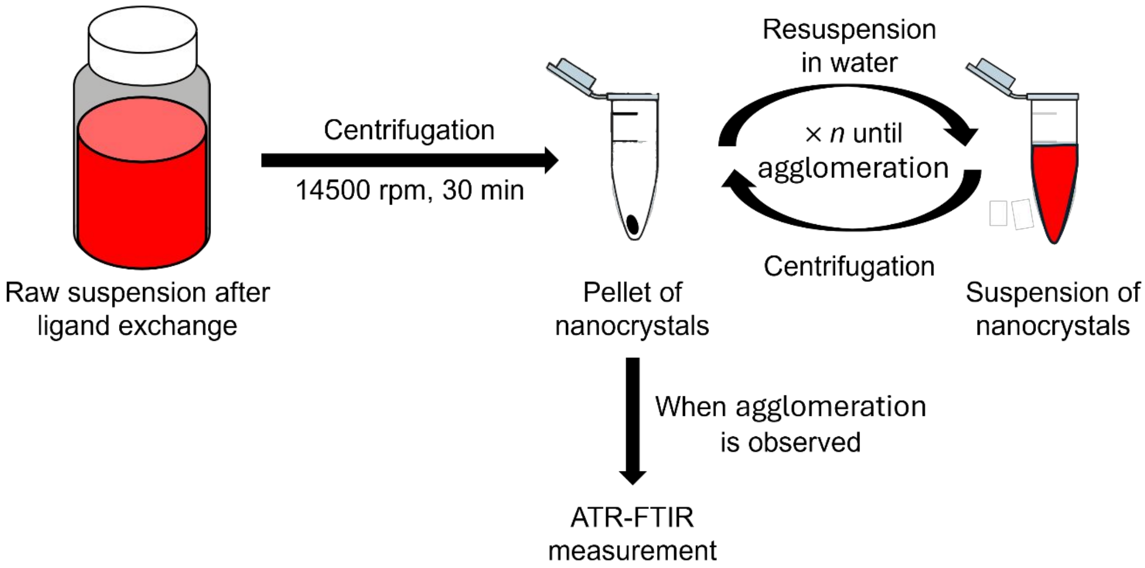

Figure S18. Schematic illustrating the procedures for removing free ligands by centrifugation and washing, as well as sample preparation for ATR-FTIR measurement.
